# Profiling and activity screening of Dammarane-type triterpen saponins from *Gynostemma pentaphyllum* with glucose-dependent insulin secretory activity

**DOI:** 10.1038/s41598-018-37517-3

**Published:** 2019-01-24

**Authors:** Lena C. E. Lundqvist, Darren Rattigan, Emad Ehtesham, Camila Demmou, Claes-Göran Östenson, Corine Sandström

**Affiliations:** 1 Department of Molecular Sciences, Swedish University of Agricultural Sciences, P. O. Box 7015, SE-750 07 Uppsala, Sweden; 2 Department of Molecular Medicine and Surgery, Endocrine and Diabetes Unit, Karolinska Institutet, Karolinska University Hospital, SE-171 76 Stockholm, Sweden; 3 Present Address: School of Medicine, The Royal College of Surgeons, Ireland 123 St Stephens Green, Dublin, 2 Ireland; 4 Present Address: Department of Public Health and Clinical Medicine, Umeå University Hospital, SE- 901 85 Umeå, Sweden

## Abstract

The global prevalence of type 2 diabetes is increasing rapidly; consequently there is great need for new and novel therapeutic options. *Gynostemma pentaphyllum (GP)* is a traditional medicinal plant, mainly present in Southeast Asian countries, that has been reported to exert antidiabetic effects, by stimulating insulin secretion. The specific compound responsible for this effect is however as yet unidentified. Screening for discovery and identification of bioactive compounds of an herbal *GP* extract, was performed in isolated pancreatic islets from spontaneously diabetic Goto-Kakizaki (GK) rats, a model of type 2 diabetes, and from non-diabetic control Wistar rats. From this herbal extract 27 dammarane-type saponins, including two novel compounds, were isolated and their structure was elucidated by mass spectrometry and NMR spectroscopy. One of the dammarane-type triterpenoid showed a glucose-dependent insulin secretion activity. This compound, gylongiposide I, displays unique abilities to stimulate insulin release at high glucose levels (16.7 mM), but limited effects at a low glucose concentration (3.3 mM). Further studies on this compound, also *in vivo*, are warranted with the aim of developing a novel anti-diabetic therapeutic with glucose-dependent insulinogenic effect.

## Introduction

*Gynostemma pentaphyllum* (Thunb.) Makino (Cucurbitaceae), *GP*, a traditional medicinal plant found in China, Vietnam, Korea and Japan, is known for containing a large amount of biologically active saponins known as dammarane-type glycosides^1,2^. The beneficial effects reported for the dammarane-type glycosides are against numerous diseases, such as cardiovascular disease, hyperlipidemia, inflammations, diabetes and cancer^2^. Currently, more than 170 unique saponins have been isolated from *GP* extracts^2–11^.

One of the dammarane-type glycosides isolated from GP extract is phanoside, a substance with identified structure and potent insulin secretory effect^12^. However, this effect is not glucose-dependent, i.e. it is stimulating insulin release at both low and high glucose concentrations^12^. Such an action can potentially induce severe hypoglycemia, similar to that of the well-known antidiabetic drug sulfonylurea. Other studies on effects of identical *GP* extract in patients with drug-naïve type 2 diabetes have demonstrated reduced glycemia and HbA1c levels, and that these effects could be attributed mainly to improved insulin sensitivity^13,14^. This was in agreement with results from a study in spontaneously type 2 diabetic Goto-Kakizaki (GK) rats, in which the *GP* extract reduced hepatic glucose output, i.e. exerted an antidiabetic effect by improving hepatic insulin sensitivity^15^. In addition, *GP* extract improved glycemic control in type 2 diabetic patients who were also treated with the sulfonylurea compound gliclazide^16^. Taken together, these findings indicate that the active component(s) in *GP* can be delivered by oral administration of the extract. However, to determine the exact mechanism of action of the extract, or the active ingredient(s) responsible for the antidiabetic effect(s), we decided to isolate and characterize the active substance(s) with focus on insulin-stimulatory effects. For that purpose, we developed a system with isolated pancreatic islets from either diabetic GK rats^15^ and healthy, control Wistar (W) rats. GK rats are non-obese and were developed by repeated breeding of W rats, selected by higher-than-normal serum glucose levels in oral glucose tolerance tests. The *GP* extract was separated by HPLC into several fractions that were tested for their potential to stimulate insulin secretion from W or GK islets at a low (3.3 mM) or a high (16.7 mM) glucose concentration. One fraction was shown to have a beneficial activity on insulin secretion and therefore the saponins comprising the fraction were further separated and their individual activity tested. By this bioassay-guided investigation of the *GP* extract, 27 saponins including two new compounds were isolated and the saponin component responsible for the biological activity of the GP extract was identified. The structures of the 27 compounds were elucidated using NMR, LC-MS and GC-MS analysis. To facilitate further studies on structural characterization of dammarane-type saponins, a library consisting of MS data, NMR ^1^H and ^13^C chemical shifts and HSQC NMR spectra was established for the 27 compounds.

## Results

**Figure 1:**
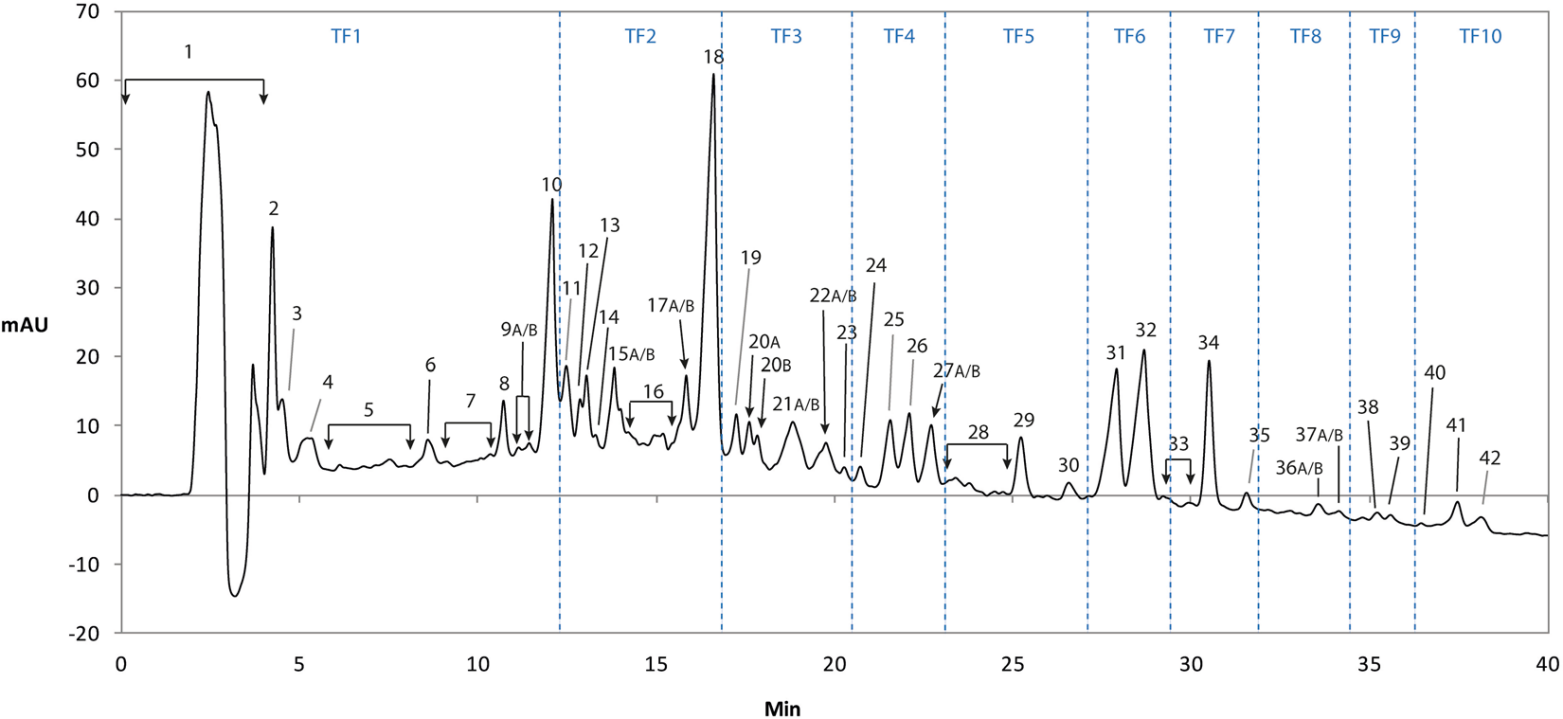
Preparative HPLC chromatogram, showing the ten sequencial time fractions TF1-TF10 as well as the fractions 1–42.

**Figure 2:**
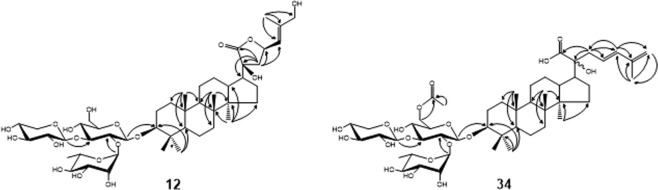
Structure of compounds **12** and **34**, and the key HMBC correlations.

In order to isolate and characterize the maximum number of saponin structures in the extract, an optimized chromatographic method was developed using a C-18 reverse-phase column (Fig. 1). The extract was fractionated by repeated preparative chromatography and the fractions were further purified by a final isocratic elution. Twenty-seven saponins were characterized and two of them, fractions **12** and **34** had new structures not previously reported. (Table 1 and Fig. 2). A compilation of the structures, NMR ^1^H and ^13^C chemical shifts and HSQC NMR spectra is available in the Supplementary Information.

**Table 1:** Saponins isolated and characterized from the GP extract.

Compound **6A**				
3β,20ξ,21ξ,23ξ-tetrahydroxy-19-oxo-21,24ξ-cyclodammar-25-ene 3-O-[α-L-rhamnopyranosyl-(1 → 2)]-[β-D-xylopyranosyl-(1 → 3)]-α-L-arabinopyranoside				
White amorphous powder	C_46_H_74_O_17_	[M + Na]^+^; calc. 921.4818	[M + Na]^+^; measured 921.4789	ref.^7^
Compound **6B**				
3*β*,20*ξ*,21*ξ*,26-tetrahydroxy-19-oxo-21,23-epoxydammar-24-ene 3-*O*-[*α*-L-rhamnopyranosyl-(1 → 2)]-[*β*-D-xylopyranosyl-(1 → 3)]-*α*-L-arabinopyranoside				
White amorphous powder	C_46_H_74_O_18_	[M + H]^+^; calc. 915.4948	[M + H]^+^; measured 915.4930	ref.^35^
Compound **8**				
(20*S*)-3*β*,20,21-trihydroxy-19-oxodammar-23,25-diene 3-*O*-[*α*-L-rhamnopyranosyl-(1 → 2)]-[*β*-D-xylopyranosyl-(1 → 3)]-*α*-L-arabinopyranosyl-21-*O*-*β*-D-glucopyranoside				
White amorphous powder	C_46_H_74_O_18_	[M + Na]^+^; calc. 1067.5397	[M + Na]^+^; measured 1067.5351	ref.^36^
Compound **10**				
3*β*,20*ξ*,21*ξ*-trihydroxy-19-oxodammar-24-ene 3-*O*-[*α*-L-rhamnopyranosyl-(1 → 2)]-[*β*-D-xylopyranosyl-(1 → 3)]-*α*-L-arabinopyranosyl-21-*O*-*β*-D-glucopyranoside				
White amorphous powder	C_52_H_86_O_21_	[M + H]^+^; calc. 1069.5554	[M + H]^+^; measured 1069.5663	ref.^26^
Compound **12**				
(3*β*,20*S*,23*R*)-3,20,23,26-tetrahydroxydammar-24-en-21-oic acid-21,23-lactone 3-*O*-[*α*-L-rhamnopyranosyl-(1 → 2)]-[*β*-D-xylopyranosyl-(1 → 3)]- *β*-D-glucopyranoside				
White amorphous powder	C_47_H_76_O_18_	[M + Na]^+^; calc. 951.4924	[M + Na]^+^; measured 951.4863	
Compound **15A**				
3*β*,20,21-trihydroxydammar-24-en 3-*O*-[*α*-L-rhamnopyranosyl-(1 → 2)]-[*β*-D-xylopyranosyl-(1 → 3)]- *β*-D-glucopyranosyl-21-*O*-*β*-D-glucopyranoside				
White amorphous powder	C_53_H_90_O_21_	[M + Na]^+^; calc. 1085.5867	[M + Na]^+^; measured 1085.5857	ref.^37^
Compound **17B**				
3*β*,20,21-trihydroxydammar-24-en 3-*O*-[*α*-L-rhamnopyranosyl-(1 → 2)]-[*β*-D-xylopyranosyl-(1 → 3)]- 6-*O*-acetyl-*β*-D-glucopyranosyl-21-*O*-*β*-D-glucopyranoside				
White amorphous powder	C_55_H_92_O_22_	[M + Na]^+^; calc. 1127.5972	[M + Na]^+^; measured 1127.5899	ref.^37^
Compound **18 (major and minor)**				
3*β*,20*ξ*,21*ξ*-trihydroxy-19-oxo-21,23-epoxydammar-24-ene 3-*O*-[*α*-L-rhamnopyranosyl-(1 → 2)]-[*β*-D-xylopyranosyl-(1 → 3)]-*α*-L-arabinopyranoside				
White amorphous powder	C_46_H_74_O_17_	[M−H_2_O]^+^; calc. 881.4893	[M−H_2_O]^+^; measured 881.4901	ref.^7^
Compound **19**				
(3*β*,20*S*,23 *S*)-3,20,23trihydroxydammar-24-en-21-oic acid-21,23-lactone 3-*O*-[*α*-L-rhamnopyranosyl-(1 → 2)]-[*β*-D-xylopyranosyl-(1 → 3)]-*α*-L-arabinopyranoside				
White amorphous powder	C_46_H_72_O_17_	[M + Na]^+^; calc. 919.4662	[M + Na]^+^; measured 919.4612	refs^21,22,35^
Compound **20A**				
(3*β*,20*S*,23*R*)-3,20,23trihydroxydammar-24-en-21-oic acid-21,23-lactone 3-*O*-[*α*-L-rhamnopyranosyl-(1 → 2)]-[*β*-D-xylopyranosyl-(1 → 3)]-*α*-L-arabinopyranoside				
White amorphous powder	C_46_H_72_O_17_	[M + Na]^+^; calc. 919.4662	[M + Na]^+^; measured 919.4625	refs^21,22^
Compound **20B**				
(3*β*,20*S*)-3,20,21-trihydroxydammar-24-ene 3-*O*-[*α*-L-rhamnopyranosyl-(1 → 2)]-[*β*-D-xylopyranosyl-(1 → 3)]-*α*-L-arabinopyranoside				
White amorphous powder	C_46_H_76_O_16_	[M + Na]^+^; calc. 907.5026	[M + Na]^+^; measured 907.4995	ref.^17^
Compound **21 (major and minor)**				
23(*R/S*)-3*β*,20*ξ*,21(*R/S*)-trihydroxy-21,23-epoxydammar-24-ene 3-*O*-[*α*-L-rhamnopyranosyl-(1 → 2)]-[*β*-D-xylopyranosyl-(1 → 3)]-*β*-D- glucopyranoside				
White amorphous powder	C_47_H_78_O_17_	[M - H_2_O]^+^; calc. 897.5206	[M - H_2_O]^+^; measured 897.5219	ref.^7^
Compound **22 (major and minor)**				
23(*R/S*)-3*β*,20*ξ*,21(*R/S*)-trihydroxy-21,23-epoxydammar-24-ene 3-*O*-[*α*-L-rhamnopyranosyl-(1 → 2)]-[*β*-D-xylopyranosyl-(1 → 3)]-*α*-L-arabinopyranoside				
White amorphous powder	C_46_H_76_O_16_	[M - H_2_O]^+^; calc. 867.5100	[M - H_2_O]^+^; measured 867.5103	ref.^7^
Compound **23**				
(3*β*,20*S*,23*S*)-3,20,23-trihydroxydammar-24-en-21-oic acid-21,23-lactone 3-*O*-[*α*-L-rhamnopyranosyl-(1 → 2)]-[*β*-D-glucopyranosyl-(1 → 3)]- *β*-D-glucopyranoside				
White amorphous powder	C_48_H_78_O_18_	[M + H]^+^; calc. 943.5261	[M + H]^+^; measured 943.5273	ref.^8^
Compound **25**				
(3*β*,20*S*,23*R*)-3,20,23-trihydroxydammar-24-en-21-oic acid-21,23-lactone 3-*O*-[*α*-L-rhamnopyranosyl-(1 → 2)]-[*β*-D-xylopyranosyl-(1 → 3)]- *β*-D-glucopyranoside				
White amorphous powder	C_47_H_76_O_17_	[M + Na]^+^; calc. 913.5155	[M + Na]^+^; measured 913.5150	refs^21,22^
Compound **26**				
(3*β*,20*S*,23*S*)-3,20,23-trihydroxydammar-24-en-21-oic acid-21,23-lactone 3-*O*-[*α*-L-rhamnopyranosyl-(1 → 2)]-[*β*-D-xylopyranosyl-(1 → 3)]- *β*-D-glucopyranoside				
White amorphous powder	C_47_H_76_O_17_	[M + Na]^+^; calc. 913.5155	[M + Na]^+^; measured 913.5144	refs^21,22^
Compound **27A**				
(3*β*,20*S*,23*R*)-3,20,23-trihydroxydammar-24-en-21-oic acid-21,23-lactone 3-*O*-[*α*-L-rhamnopyranosyl-(1 → 2)]-[*β*-D-xylopyranosyl-(1 → 3)]- *α*-L-arabinopyranoside				
White amorphous powder	C_46_H_74_O_16_	[M + Na]^+^; calc. 905.4869	[M + Na]^+^; measured 905.4877	refs^21,22^
Compound **27B**				
(3*β*,20*S*,23*S*)-3,20,23-trihydroxydammar-24-en-21-oic acid-21,23-lactone 3-*O*-[*α*-L-rhamnopyranosyl-(1 → 2)]-[*β*-D-xylopyranosyl-(1 → 3)]- *α*-L-arabinopyranoside				
White amorphous powder	C_46_H_74_O_16_	[M + Na]^+^; calc. 905.4869	[M + Na]^+^; measured 905.4877	refs^21,22^
Compound **29**				
3*β*,20-dihydroxydammar-23,25-diene-21-carboxylic acid 3-*O*-[*α*-L-rhamnopyranosyl-(1 → 2)]-[*β*-D-xylopyranosyl-(1 → 3)]-*β*-D-glucopyranoside				
White amorphous powder	C_47_H_76_O_17_	[M + Na]^+^; calc. 935.4975	[M + Na]^+^; measured 935.4961	ref.^38^
Compound **31**				
(3*β*,20*S*,23*R*)-3,20,23-trihydroxydammar-24-en-21-oic acid-21,23-lactone 3-*O*-[*α*-L-rhamnopyranosyl-(1 → 2)]-[*β*-D-xylopyranosyl-(1 → 3)]-6-*O*-acetyl-*β*-D-glucopyranoside				
White amorphous powder	C_49_H_78_O_18_	[M + Na]^+^; calc. 977.5080	[M + Na]^+^; measured 977.5033	ref.^8^
Compound **32**				
(3*β*,20*S*,23*S*)-3,20,23-trihydroxydammar-24-en-21-oic acid-21,23-lactone 3-*O*-[*α*-L-rhamnopyranosyl-(1 → 2)]-[*β*-D-xylopyranosyl-(1 → 3)]-6-*O*-acetyl-*β*-D-glucopyranoside				
White amorphous powder	C_49_H_78_O_18_	[M + Na]^+^; calc. 977.5080	[M + Na]^+^; measured 977.5021	ref.^8^
Compound **34**				
3*β*,20-dihydroxydammar-23,25-diene-21-carboxylic acid 3-*O*-[*α*-L-rhamnopyranosyl-(1 → 2)]-[*β*-D-xylopyranosyl-(1 → 3)]-*β*-D-6-*O*-acetylglucopyranoside				
White amorphous powder	C_49_H_78_O_18_	[M + Na]^+^; calc. 977.5080	[M + Na]^+^; measured 977.5047	
Compound **39**				
(3*β*,20*S*,23*S*)-3,20,23-trihydroxydammar-24-en-21-oic acid-21,23-lactone 3-*O*-[*α*-L-rhamnopyranosyl-(1 → 2)]-[*β*-D-xylopyranosyl-(1 → 3)]-6-*O*-acetyl-*β*-D-glucopyranoside				
White amorphous powder	C_51_H_80_O_19_	[M + Na]^+^; calc. 1019.5186	[M + Na]^+^; measured 1019.5140	ref.^8^
Compound **41**				
(23*ξ*)-21*ξ*-*O*-*n*-butyl-3*β*,20*ξ*,21*ξ*-trihydroxy-19-oxo-21,23-epoxydammar-24-ene 3-*O*-[*α*-L-rhamnopyranosyl-(1 → 2)]-[*β*-D-xylopyranosyl-(1 → 3)]-*α*-L-arabinopyranoside				
White amorphous powder	C_50_H_82_O_17_	[M + Na]^+^; calc. 977.5444	[M + Na]^+^; measured 977.5407	ref.^39^

### Biological activity

**Figure 3:**
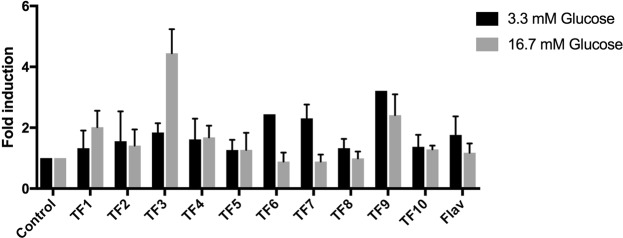
Result of the screening of the time fraction for biological activity by incubating the extract of the ten time fraction with pancreatic islets of either W or GK rats to determine effects on insulin release at both low (3.3 mM) and high (16.7 mM) glucose concentrations.

**Figure 4:**
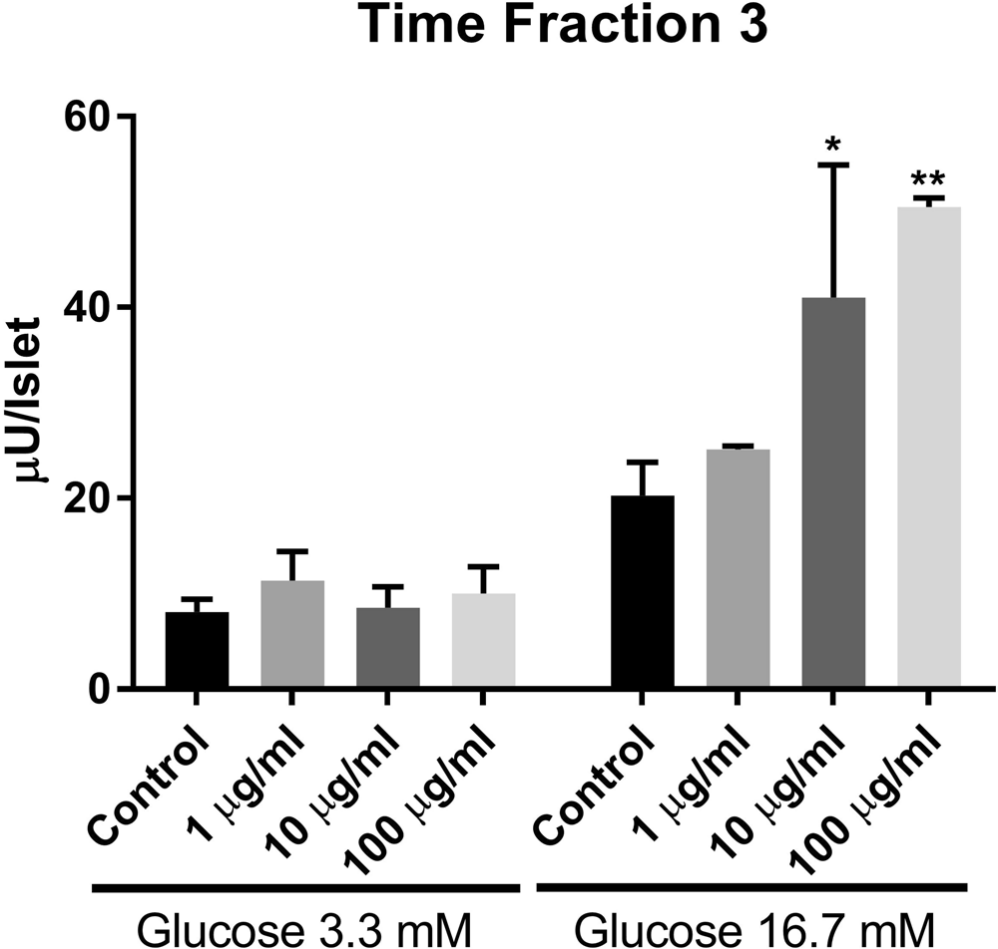
Effects of time fraction 3 (1, 10 and 100 µg/ml) on insulin release from isolated rat islets at either 3.3 or 16.7 mM glucose. *p < 0.05; **p < 0.01 or less.

To locate the active saponins, ten sequential time fractions (Fig. 1) from the HPLC separation were collected and screened for biological activity. GP extract fractions or compounds isolated from the fractions were incubated with pancreatic islets of either W or GK rats to determine effects on insulin release at low (3.3 mM) and high (16.7 mM) glucose concentrations. No significant insulin stimulatory activity was found in TF1-TF2, TF4-TF8 and TF10, or the flavonoid fraction (Fig. 3), while such an activity was found in TF3 and TF9. TF9 enhanced insulin release to a similar extent regardless of in the presence of 3.3 or 16.7 mM glucose. TF3 dose-dependently increased insulin release at 16.7 mM glucose, while it did not enhance insulin release at 3.3 mM glucose (Fig. 4).

**Figure 5:**
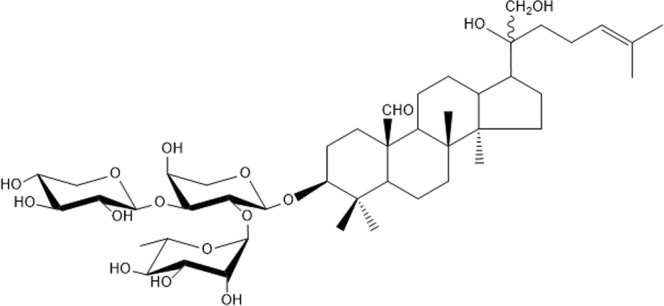
Structure of compound **20B** (gylongiposide I).

**Figure 6:**
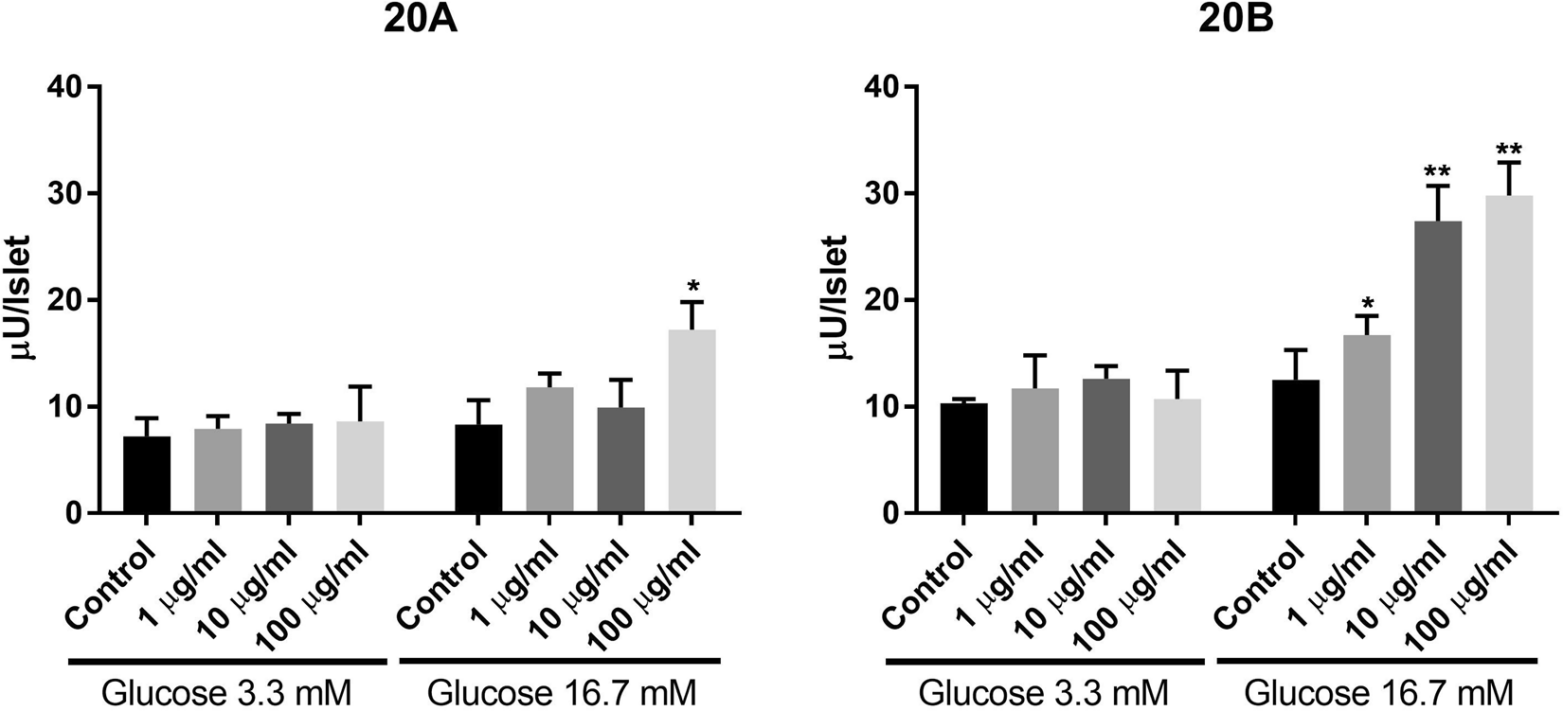
Effects of compounds **20A** and **20B** (1, 10 and 100 µg/ml) on insulin secretion at 3.3 and 16.7 mM glucose, from batch-incubated pancreatic islets isolated from spontaneously diabetic Goto-Kakizaki (GK) rats. *p < 0.05; **p < 0.01 or less.

The saponins in fractions TF3 were further isolated and tested for their individual activity. Two compounds **20A** and **20B** in TF3 were found to stimulate insulin secretion. Compound **20B** (Fig. 5) showed beneficial biological activity by only stimulating the insulin secretion at high glucose concentration (Fig. 6). This compound (**20B**) known as gylongiposide I has been isolated and its structure reported previously but has not been studied for its anti-diabetic activity^17–20^.

### Structural elucidation

Compound **12** (Fig. 2) was obtained as a white, amorphous powder, the molecular formula was established as C_47_H_76_O_18_ and a molecular ion peak was observed by both positive and negative-mode ESI-MS (*m/z* 951.4863 [M + Na]^+^; calc. 951.4924). The ^1^H-^13^C multiplicity edited HSQC NMR (CD_3_OD, 600 MHz) spectrum of **12** indicated the presence of seven methyl signals at *δ*_H_ 0.86, 0.89, 0.93, 1.03, 1.03, 1.21, 1.75, twelve methylene signals, and twenty-one methine signals.

The NMR data of the aglycone moiety (Supporting Information S5e) showed characteristic resonances of carbons from one lactone carbonyl carbon *δ*_c_ 180.43, one double bond (*δ*_C_ 123.96, 142.68). COSY and TOCSY cross-peaks of H_2_-1/H_2_-2/H-3; H-5/H_2_-6/H_2_-7; H-9/H_2_-11/H_2_-12/H-13/H-17/H_2_-16/H_2_-15 and H_2_-22/H-23/H-24 demonstrated the presence of vicinal coupling system. In the HMBC spectrum, two and three bond correlations of H_3_-18/C-7, C-8, C-9, and C-14; H-19/C-1, C-5, C-9, and C-10; H_3_-27/C-24, C-25 C-26; H_3_-28, H_3_-29/C-3, C-4, and C-5; H_3_-30/C-8, C-13, C-14, and C-15; H_2_-22/C-17, C-20, C-21, C-23, and C-24, together with the chemical shift of all protons and carbons, reveal that compound **12** had a 3,20,23,26-tetrahydroxydammar-24-en-21-oic acid-21,23-lactone aglycone structure.

The NMR data of the glycoside moiety (Supporting Information S5e) showed three anomeric signals, at *δ*_H/C_ 4.44/105.08 (H1/C1 of Glc), *δ*_H/C_ 4.46/104.79 (H1/C1 of Xyl), and *δ*_H/C_ 5.42/101.70 (H1/C1 of Rha), two hydroxymethyl groups H6/C6 of Glc (3.67, 3.85/62.47) and H5/C5 of Xyl (3.28, 3.93/67.01), one methyl group C6 of Rha (1.21/17.88) and eleven oxygen-bearing sugar carbons and their attached protons in the region *δ*_C_ 69.94–87.95, *δ*_H_ 3.26–3.98. Three spin systems were identified through TOCSY experiment. The configuration was determined to be in the α and β form based on the magnitude of the H1-H2 coupling constant *β*-D-*Xyl* (H1, d, ^3^*J* = 7.6 Hz), *β*-D-*Glc* (H1, d, ^3^*J* = 7.6 Hz), and *α*-L-*Rha* (H-1, ^3^*J* = 1.5). HMBC correlations (Fig. 2) between H-1′′ *Rha* (*δ*_H_ 5.42) and C-2′ *Glc* (*δ*_C_ 77.85), H-1′′′ *Xyl* (*δ*_H_ 4.46) and C-3′ *Glc* (*δ*_C_ 87.95), H-1′ *Glc* (*δ*_H_ 4.44) and C-3 on the aglycone (*δ*_C_ 89.85), demonstrated that the *β*-D-glucopyranosyloxy unit was located at C-3 of the aglycone and that the *α*-L-rhamnopyranosyloxy and *β*-D-xylopyranosyloxy units were linked to C-2′ and C-3′ of the glucopyranosyloxy unit respectively.

The configuration at C-20 and C-23 were determined by comparison with the most recent studies of configuration at C-20 and C-23 carried out by electronic circular dichroism (ECD) and X-ray diffraction analysis^21,22^. Therefore, compound **12** was assigned to be (3*β*,20*S*,23*R*)-3,20,23,26-tetrahydroxydammar-24-en-21-oic acid-21,23-lactone 3-*O*-[*α*-L-rhamnopyranosyl-(1 → 2)]-[*β*-D-xylopyranosyl-(1 → 3)]- *β*-D-glucopyranoside.

Compound **34** (Fig. 2) was obtained as a white, amorphous powder, the molecular formula was established as C_49_H_78_O_18_ and a molecular ion peak was observed by positive-mode ESI-MS (*m/z* 977.5047 [M + Na]^+^; calc. 977.5080). The ^1^H-^13^C multiplicity edited HSQC NMR (CD_3_OD, 600 MHz) spectrum of **34** indicated the presence of eight methyl signals at *δ*_*H*_ 0.85, 0.87, 0.89, 0.98, 1.03, 1.21, 1.79, 2.05, four olefin signals, eleven methylene signals, and nineteen methine signals.

The NMR data of the aglycone moiety (Supporting Information S21e) showed characteristic resonances of one double bond (*δ*_C_ 126.38, 136.79), one olefin carbon (*δ*_C_ 114.97) arising from the double bond between C-25 and C-26. COSY and TOCSY cross-peaks of H_2_-1/H_2_-2/H-3; H-5/H_2_-6/H_2_-7; H-9/H_2_-11/H_2_-12/H-13/H-17/H_2_-16/H_2_-15 and H_2_-22/H-23/H-24 demonstrated the presence of vicinal coupling system. In the HMBC spectrum, two and three bond correlation of H_3_-18/C-7, C-8, C-9 and C-14; H-19/C-1, C-5 and C-10; H_2_-26/C-27, C-24 and C-25; H_3_-27/C-24, C-25 and C-26; H_3_-28, H_3_-29/C-3, C-4 and C-5; H_3_-30/C-14 and C-15; H_2_-22/C-20, C-21, C-23 and C-24; H-24/C-22, C-25 and C-26, together with the chemical shift of all protons and carbons, reveal that **34** had a 3,20-dihydroxydammar-23,25-diene-21-carboxylic acid aglycone structure.

The NMR data of the glycoside moiety (Supporting Information S21e) showed three anomeric signals, at *δ*_H/C_ 4.44/105.01 (H1/C1 of Glc), *δ*_H/C_ 4.45/104.87 (H1/C1 of Xyl), and *δ*_H/C_ 5.41/101.79 (H1/C1 of Rha), two hydroxymethyl groups H6/C6 of Glc (4.23, 4.37/64.60) and H5/C5 of Xyl (3.27, 3.93/67.01), one methyl group C6 of Rha (1.21/17.97) and eleven oxygen-bearing sugar carbons and their attached protons in the region *δ*_C_ 70.16–87.73, *δ*_H_ 3.26–3.99. Three spin systems were identified through TOCSY experiment. An acetyl group at *δ*_H_ 2.05 and *δ*_C_ 172.33, 20.76, showed HMBC correlation between H-6 of Glc and the carbonyl carbon of this acetyl group. The configuration were determined to be in the α and β form based on the coupling constant *β*-D-*Xyl* (H1, d, ^3^*J* = 7.3 Hz), *β*-D-*Glc* (H1, d, ^3^*J* = 7.6 Hz), and *α*-L-*Rha* (H-1, ^3^*J* = 1.30). HMBC correlations (Fig. 2) between H-1′′ *Rha* (*δ*_H_ 5.41) and C-2′ *Glc* (*δ*_C_ 77.92), H-1′′′ *Xyl* (*δ*_H_ 4.45) and C-3′ *Glc* (*δ*_C_ 87.73), H-1′ *Glc* (*δ*_H_ 4.44) and C-3 on the aglycone (*δ*_C_ 90.44), demonstrated that the *β*-D-6-*O*-acetylglucopyranosyloxy unit was located at C-3 of the aglycone and that the *α*-L-rhamnopyranosyloxy and *β*-D-xylopyranosyloxy units were linked to C-2′ and C-3′ of the glucopyranosyloxy unit respectively.

Compound **34** were assigned to be 3*β*,20-dihydroxydammar-23,25-diene-21-carboxylic acid 3-*O*-[*α*-L-rhamnopyranosyl-(1 → 2)]-[*β*-D-xylopyranosyl-(1 → 3)]-*β*-D-6-*O*-acetylglucopyranoside.

## Discussion

### Biological activity

We have identified and characterized the dammarane-type triterpene saponin responsible for the insulin secretion induced by a Chinese extract of the *Gynostemma Pentaphyllum* (*GP*) herbal plant. Although several of the pooled time fractions, TFs, obtained from the HPLC chromatogram, had varying degree of effect on insulin secretion from isolated islets of control W and diabetic GK rats, significant effects on the insulin-producing beta-cells were seen with one of the fractions, TF3. In addition, we found that TF3 had the most favourable antidiabetic effect by enhancing insulin release significantly only at high glucose but not at low glucose concentrations. This is in contrast to the well-known antidiabetic sulfonylurea drugs, that stimulate insulin secretion also at normoglycemia or even at lower glucose levels^23^ and thereby can cause serious hypoglycemia in patients. By further chromatographic separation of the saponins present in TF3 followed by bio-assay testing we were able to determine that this favourable activity, i.e. a glucose-dependent stimulation of insulin release was entirely related to the saponin compound **20B**.

In this context, it is of great importance to note that this compound most likely retains its biological activity following oral administration, since we have shown prominent antidiabetic activity in diabetic GK rats, treated orally with GP extract (0.3 g/kg body weight) for 2 weeks^24^. In these animals, the improvement of glucose tolerance was demonstrated in parallel with augmented plasma insulin levels. A similar effect was previously reported by another compound isolated from GP extract, i.e. phanoside^12,25^. However, in contrast to the present saponin **20B**, phanoside did not stimulate insulin secretion in a glucose-dependent manner. Recent mechanistic studies on the GP extract demonstrated that the stimulation of insulin secretion from rat islets was mediated by effects on several steps in the stimulus-secretion coupling to glucose in the pancreatic beta cells^24^. Among these steps are the K-ATP channels, the L-type Ca^2+^ channels, the protein kinase A activity, and pertussis-toxin sensitive exocytotic G_e_-proteins.

### Structure and activity

*Gynostemma pentaphyllum* extracts are known to possess numerous biological activities through their dammarane-type triterpene saponins. The possible structural-activity relationships (SAR) is related to the number and nature of the sugars, their acylation, the types of aglycones and the stereochemistry. A large number of studies have been conducted on anti-tumor, anti-inflammatory and anti-diabetic activities, where different structural components have been pointed out as important for the activities^2,24,26^. The presence of a hydroxyl group at position C-3^9^, a double bond between C20-C22 or C20-C21^27^, and a five membered unsaturated ketone in the side chain^28^ in the dammarane-type structure has been mentioned as possible structural components responsible for anti-tumor activity. These results are from studies carried out on different cancer cell lines and are therefore difficult to compare. The differences in anti-inflammatory abilities have been explained by differences in sugar moieties^29^.

**Figure 7:**
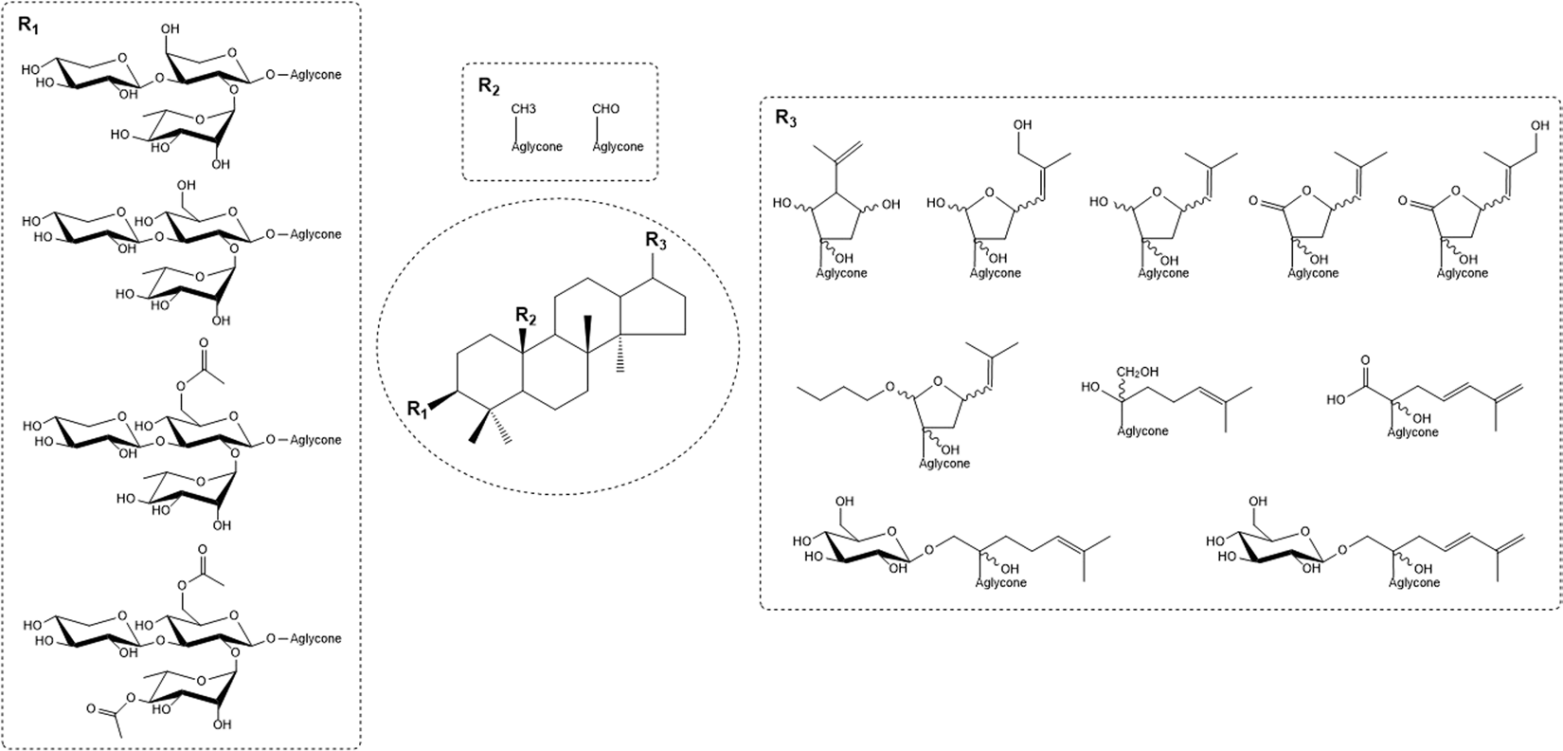
Structural components of the dammarane-type saponins isolated and characterized in the GP extract. The chemical structures of the 27 isolated compounds are reported in the Supporting Information S1a–S23a.

From the present work, some structural features are worth noticing: The glycoside moiety at the C-3 position on the aglycone of the isolated saponins consists of a branched trisaccharide either *α*-L-Rha-(1 → 2)-[*β*-D-Xyl-(1 → 3)]-*α*-L-Ara, or *α*-L-Rha-(1 → 2)-[*β*-D-Xyl-(1 → 3)]-*β*-D-Glc. More seldom *α*-L-Rha-(1 → 2)-[*β*-D-Xyl-(1 → 3)]-*β*-D-Glc with acetylation on C6 of glucose and in some cases also on C4 of rhamnose. In some cases, as in compounds **8**, **10**, **15** and **17** an additional glucopyranose unit is attached at C-21. The aglycone moiety shows structural variability at two positions. The substituent at C-19 can be either a methyl or an aldehyde group. The greatest variability between the structures is found in the side chain attached to C-17. Ten different side chains attached to C-17 were found, some composed of five membered rings, either as lactone or hemiacetal, other as open structures with different number of double bonds and/or additional glycoside moiety attached at C-21 (Fig. 7).

Interestingly, the potential to stimulate insulin secretion at high glucose concentration is limited to one single compound, **20B**, while several saponins can stimulate insulin secretion independent of glucose concentration. Comparison of the structures of the different saponins shows that compound **10** has a structure very similar to that of **20B** with the same trisaccharide unit at C-3, an aldehyde group at C-19 and the same open chain attached at C-17 with a double bond between C-24 and C-25. The only difference between the two compounds is the presence of an additional glucose residue at C-21 in compound **10**. Thus, the presence of an additional sugar appears to suppress the insulin secretion at high glucose concentration.

Because of the increasing amount of data on the biological activities of saponins, rapid screening methods including activity and structural data such as LC-MS and NMR are valuable^30^. The ^1^H-^13^C HSQC NMR spectra of the 27 identified saponins together with the MS data and ^1^H and ^13^C NMR chemical shifts reported in the present work should help in structural analysis when investigating new extract, and may allow for more rapid screening of already known compounds.

## Experimental Procedures

### Material and reagents

*Gynostemma Pentaphyllum* (*GP*) herbal extract was purchased from Hanzhong TRG Biotech Co.,Ltd. Solid phase extraction column Hypersep C-18 was purchased from Thermo scientific. Deionized water was purified by Milli-Q system from Millipore. Ethanol absolute 100% Ph.Eur, acetonitrile (HPLC grade) and methanol (HPLC grade) were purchased from VWR chemicals. Methanol D4 99.80% D (NMR) were purchased from Euriso-top. Collagenase, HEPES, bovine albumin, D-glucose, L-glutamine, RPMI 1640 culture medium, and fetal calf serum were obtained from Sigma-Aldrich, Sweden.

### Extraction and isolation

The dried and powdered *GP* extract was dissolved in 30% ethanol solution, and applied to a Hypersep C-18 cartridge. The column was washed with 30% ethanol solution (flavonoid fraction). The saponin fraction was collected by elution with 100% ethanol. The saponin fractions were dried and further separated through preparative HPLC (Gilson 305/306 system) on a 20 × 150 mm C-18 column (Kromasil 100-5C18 HiCHROM, UK) with a binary solvent system of an aqueous (Milli-Q) 0.1% formic acid solution (A) and acetonitrile (B), with the following gradient elution: initially 30% (B) – 70% (A), changed to 50% (B) after 7 min, 50% (B) to 15 min, 60% (B) to 35 min, 80% (B) to 40 min and returned to 30% (B) after 41 min. The detection (Gilson 118 UV/VIS detector) was done at 210 nm, and a flow rate of 10 ml/min was used.

Ten sequential time fractions from the HPLC separation were collected (Fig. 1), lyophilized and stored at 4 °C until activity testing.

Furthermore, fractions 1–42 (Fig. 1) were collected and lyophilized, through repeated injection to receive sufficient material for NMR analysis to determine if the fractions contain flavonoids, saponins or other components. Some of the 42 screened compounds were not saponins and are therefore not reported. Enough material were collected through repeated injections for exhaustive structural analysis of 27 saponins fractions. The fractions were then lyophilized and purified by isocratic elution according table S24 available in the Supporting Information. Fractions **20A** and **20B** were co-eluting and separated by an isocratic elution (50:50) of a binary solvent system of an aqueous 0.1% formic acid solution (A) and 80:20% acetonitrile:methanol (B). The purified fractions were stored at 4 °C until NMR and LC-MS analysis.

### NMR

The NMR data were recorded at 298 K with a Bruker AVANCE™ III 600 MHz spectrometer using a 5 mm ^1^H/^13^C/^15^N/^31^P cryoprobe or a 5 mm broadband observe detection SmartProbe, both equipped with z-gradient. The ^13^C and ^1^H chemical shifts were measured using CD_3_OD as an internal standard (*δ* = 3.31 and 49.15 ppm for proton and carbon respectively). The data were acquired and processed using Bruker software. The ^1^H and ^13^C resonances were assigned using homonuclear ^1^H–^1^H COSY, TOCSY and NOESY and heteronuclear ^1^H–^13^C HSQC, HSQC–TOCSY and HMBC experiments from the Bruker pulse sequence library.

### LC-MS

The LCMS analysis were carried out on isolated fractions from the crude extract with an Agilent 1100 liquid chromatography system equipped with a variable UV wavelength detector and a Kromasil^®^ C-18 reverse-phase (4.6 mm × 150, 5 µm) column using suitable isocratic condition for each isolated fraction of aqueous:organic solvent with the inclusion of 0.1% formic acid into both aqueous (Milli-Q) and organic phase in which the later phase consists of acetonitrile and methanol 8:2 (v/v). The flow rate was set at 1 ml/min and the UV detection wavelength was set at 210 nm to verify and screen UV peak of the compounds with their respective total ion chromatogram (TIC) peak.

The accurate mass analysis on the saponins were determined using Maxis Impact Bruker^®^ MS-QTOF coupled to the LC system and using Compass^®^ Bruker data processing software.

### Animals and pancreatic islet isolation

Male diabetic GK rats and control Wistar (W) rats were used. The GK rats were bred within the department^31^, while the W rats were purchased from Charles River Ltd. The rats were maintained in a 12 h light and dark cycle and allowed food and water ad libitum. The GK rats (n = 25) had body weights ranging from 160 to 277 g, and their non-fasted plasma glucose levels were 6.4–10.9 mM, while W rats (n = 8) were 199–267 g with plasma glucose levels 4.4–5.9 mM. The animals were sacrificed by CO_2_ to allow removal of pancreata for islet isolation by collagenase digestion as described^32^. Isolated islets were handpicked under a stereo microscope, maintained for 24 h at 37 °C and 5% CO_2_ in culture dishes with RPMI 1640 medium, containing 10% heat-inactivated fetal calf serum, 2 mM L-glutamine, and 11 mM glucose prior to further experiments with incubations to determine insulin secretion^25,31,33^. All methods and experimental protocols were carried out in concert with relevant guidelines and regulations. In addition, the studies were performed after approval by the laboratory animal ethics committee of the Karolinska Institutet, Stockholm.

### Islet batch incubations

Isolated islets were pre-incubated for 45 min in Krebs-Ringer bicarbonate (KRB) buffer^25,33^, pH 7.4, and supplemented with 10 mM HEPES, 0.2% bovine albumin and 3.3 mM glucose. Thereafter, batches of three islets were incubated in KRB buffer with 10 mM HEPES, 0.2% albumin and either 3.3 or 16.7 mM glucose. In addition, *GP* fractions were filtered through a 0.20 μm syringe filter prior to the incubations to remove any debris from the sample. The *GP* fractions were diluted to final concentrations of 100 µg/ml, 10 µg/ml, 1 µg/ml, and a control of 0 µg/ml in buffer with either glucose concentration. Each *GP* fraction was tested in triplicates, and each batch of islets was incubated for 60 min in 300 µl buffer in a shaking water bath at 37 °C, and then 200 µl of the incubation media was removed and stored at −18 °C until later determination of insulin. Experiments were repeated two times, and hence there were 3–6 observations from each fraction. Insulin was determined by radioimmunoassay as described^34^.

### Statistical analysis

All results from insulin secretion experiments were analysed and presented as mean and standard error of the mean (SEM): One-way Anova was performed to evaluate statistical significance of results. When this analysis indicated statistical significance, the results were further analysed by Fisher’s least significant difference. Statistical analysis was conducted on each of the individual batch incubations and therefore n = 3–6 for each of the fractions tested at each concentration. The n value varied depending on the number of replicas of the experiment conducted with triplicates. A p value < 0.05 was considered statistically significant. To allow comparisons between the groups, the mean results for each concentration of the pooled fraction, or pure saponin, were divided by their respective control at low and high glucose levels. This allowed a direct comparison between the groups since the absolute values of insulin secretion from the islets varied between rats. The results were therefore presented as fold induction of insulin release compared against 3.3 mM or 16.7 mM glucose control values.

## Supplementary information

Supplementary Information
