# Supplementary material for: Profiling and activity screening of Dammarane-type triterpen saponins from Gynostemma pentaphyllum with glucose-dependent insulin secretory activity
Source: Sci Rep. 2019 Jan 24;9:627. doi: 10.1038/s41598-018-37517-3 (PMC6345837; doi:10.1038/s41598-018-37517-3)
Supplement: Supplementary file 1 — Supplementary Information [file 41598_2018_37517_MOESM1_ESM.pdf]

Profiling and activity screening of Dammarane-type triterpen saponins from *Gynostemma pentaphyllum* with glucose-dependent insulin secretory activity.

Lena C. E. Lundqvist[a,*], Darren Rattigan[b,+], Emad Ehtesham[a,#], Camila Demmou[a], Claes-Göran Östenson[b,*] and Corine Sandström[a]

[a]Department of Molecular Sciences, Swedish University of Agricultural Sciences, P. O. Box 7015, SE-750 07 Uppsala, Sweden

[b]Department of Molecular Medicine and Surgery, Endocrine and Diabetes Unit, Karolinska Institutet, Karolinska University Hospital, SE-171 76 Stockholm, Sweden

[+]Present address: School of Medicine, The Royal College of Surgeons Ireland, 123 St Stephens Green, Dublin 2, Ireland.

[#]Present address: Department of Public Health and Clinical Medicine, Umeå University Hospital, SE-901 85 Umeå, Sweden

[*] Shared authorship

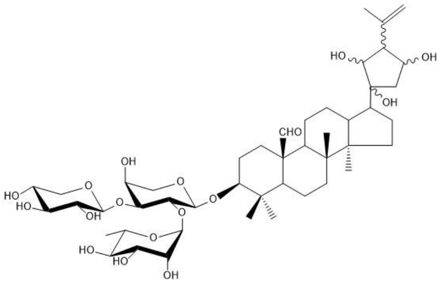

S1a. Structure of compound 6A.

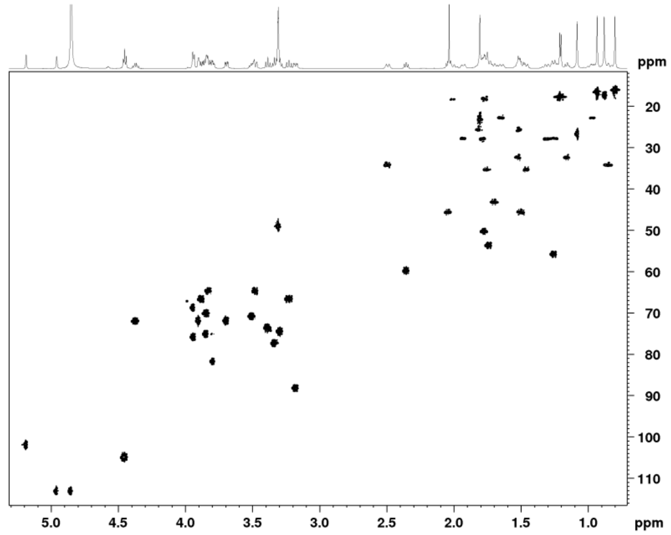

S1b. HSQC of compound 6A.

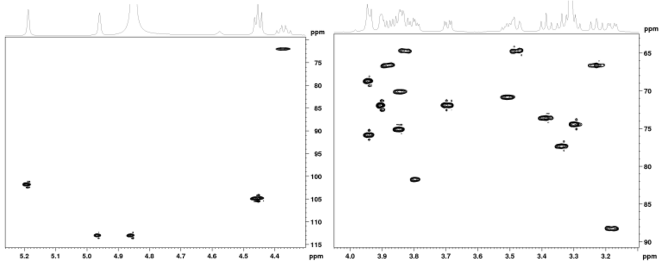

S1c. Selected region of HSQC showing anomeric and ringproton of the glycoside moiety of compound 6A.

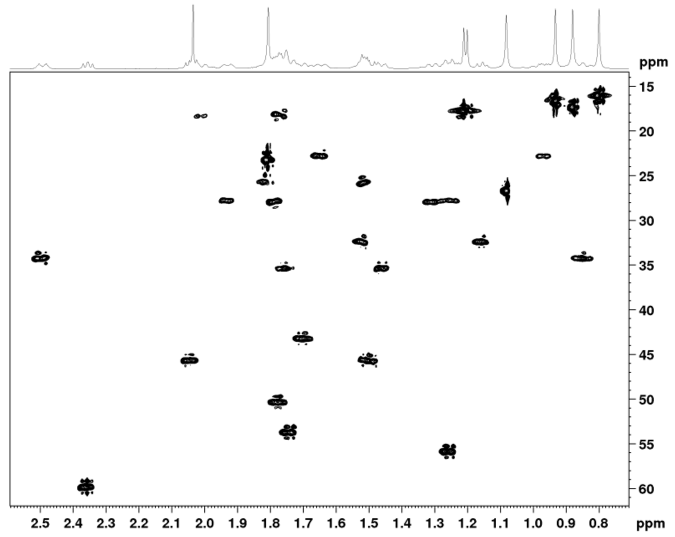

S1d. Selected region of HSQC showing the aglycone moiety of compound 6A.

S1e. Chemical shifts of compound 6A.

Pos.	6A	
	δH	δC
1	0.85, 2.50	34.28
2	1.32, 1.78	27.94
3	3.18	88.24
4	------	40.65
5	1.26	55.89
6	1.77, 2.01	18.40
7	1.46, 1.75	35.39
8	------	41.20
9	1.75	53.69
10	------	53.66
11	0.97, 1.65	22.80
12	1.52, 1.82	25.88
13	1.70	43.30
14	------	50.55
15	1.16, 1.52	32.47
16	1.25, 1.93	27.78
17	1.78	50.33
18	0.89	17.39
19	10.18	207.79
20	------	81.12
21	3.94	75.81
22	1.50, 2.05	45.65
23	4.37	72.00
24	2.36	59.85
25	------	143.23
26	4.86, 4.96	113.14
27	1.82	23.24
28	1.08	26.76
29	0.81	16.07
30	0.93	16.65
	Ara	
1'	4.46	104.95
2'	3.85	75.07
3'	3.80	81.66
4'	3.94	68.77
5'	3.48, 3.83	64.75
	Rha	
1''	5.19	101.86
2''	3.91	72.00
3''	3.70	72.00
4''	3.39	73.61
5''	3.85	70.10
6''	1.21	17.83
	Xyl	
1'''	4.44	104.79
2'''	2.29	74.48
3'''	3.34	77.27
4'''	3.51	70.85
5'''	3.23, 3.88	66.65

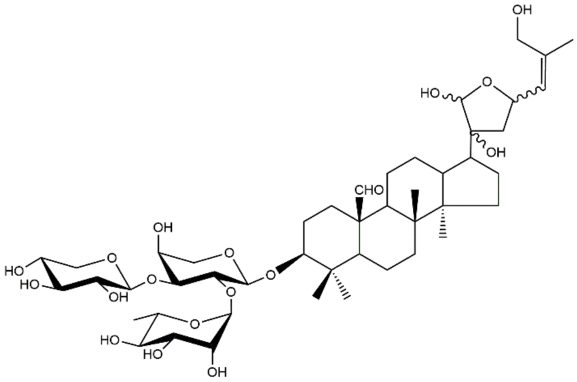

S2a. Structure of compound 6B.

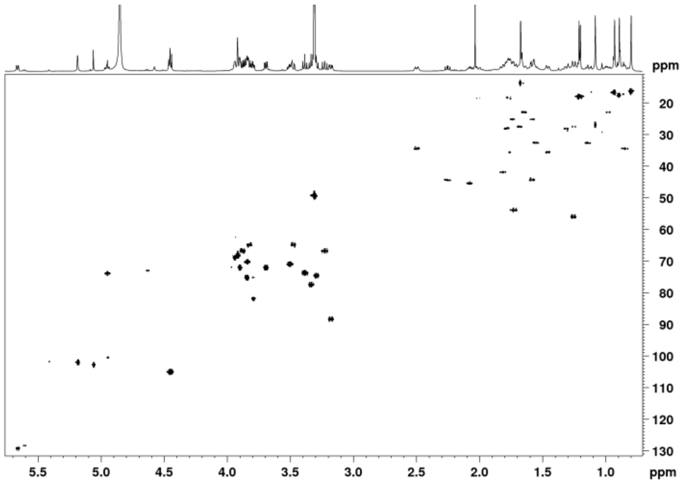

S2b. HSQC of compound 6B.

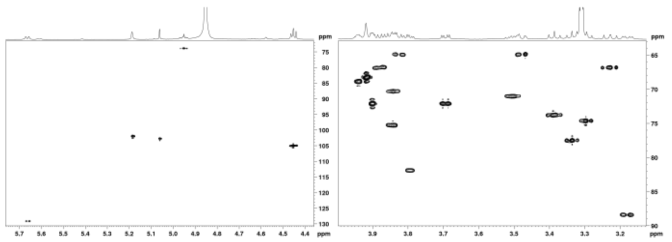

S2c. Selected region of HSQC showing anomeric and ringproton of the glycoside moiety of compound 6B.

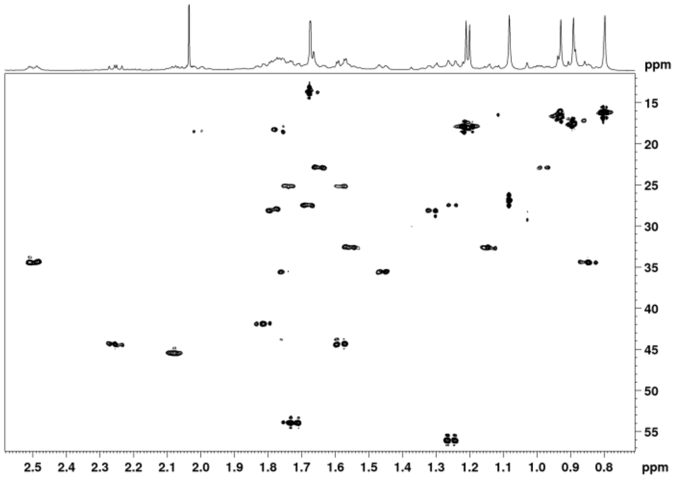

S2d. Selected region of HSQC showing the aglycone moiety of compound 6B.

S2e. Chemical shifts of compound 6B.

Pos.	6B	
	δH	δC
1	0.85, 2.50	34.36
2	1.31, 1.79	28.07
3	3.18	88.38
4	------	40.81
5	1.25	56.02
6	1.77, 2.00	18.54
7	1.46, 1.76	35.53
8	------	41.23
9	1.73	53.90
10	------	53.96
11	0.98, 1.64	22.94
12	1.58, 1.74	25.13
13	1.82	41.83
14	------	50.66
15	1.14, 1.54	32.60
16	1.25, 1.68	27.48
17	2.08	45.49
18	0.89	17.82
19	10.18	207.93
20	------	85.37
21	5.06	102.72
22	1.58, 2.25	44.38
23	4.95	73.88
24	5.67	129.23
25	------	137.44
26	3.92	68.32
27	1.68	13.86
28	1.08	26.88
29	0.80	16.20
30	0.92	16.64
	Ara	
1'	4.45	105.08
2'	3.84	75.21
3'	3.80	81.86
4'	3.94	68.91
5'	3.47, 3.83	64.88
	Rha	
1''	5.18	102.00
2''	3.90	72.06
3''	3.69	72.13
4''	3.39	73.74
5''	3.84	70.22
6''	1.21	17.96
	Xyl	
1'''	4.44	105.08
2'''	3.29	74.62
3'''	3.33	77.40
4'''	3.51	70.96
5'''	3.22, 3.88	66.79

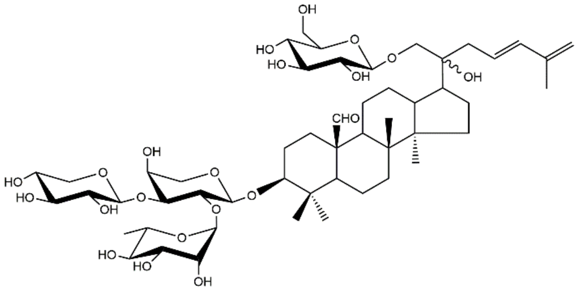

S3a. Structure of compound 8.

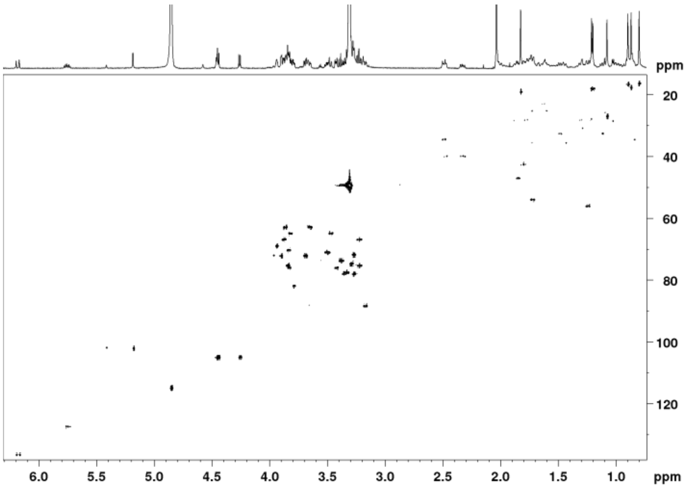

S3b. HSQC of compound 8.

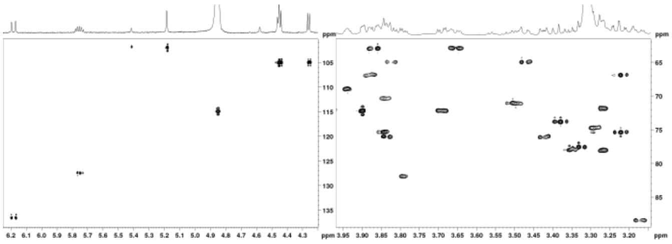

S3c. Selected region of HSQC showing anomeric and ringproton of the glycoside moiety of compound 8.

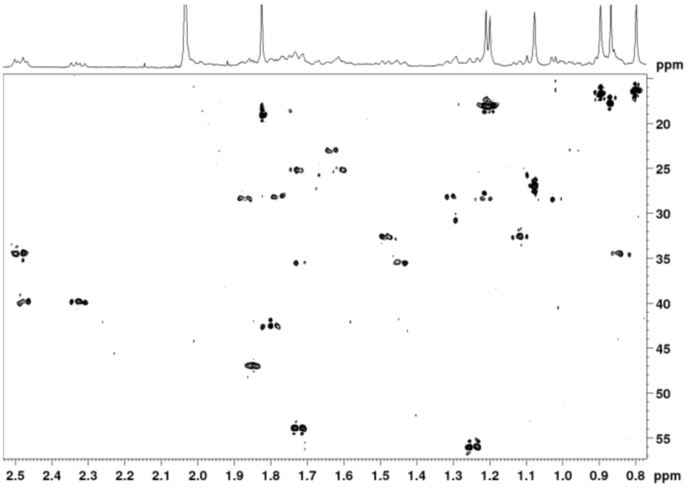

S3d. Selected region of HSQC showing the aglycone moiety of compound 8.

S3e. Chemical shifts of compound 8.

Pos.	8	
	δH	δC
1	0.84,2.49	34.51
2	1.31, 1.78	28.07
3	3.18	88.38
4	------	40.72
5	1.25	56.04
6	1.75, 1.99	18.55
7	1.44, 1.72	35.39
8	------	41.64
9	1.72	53.89
10	------	53.85
11	0.97, 1.64	22.93
12	1.62, 1.73	25.13
13	1.80	42.48
14	------	51.03
15	1.12, 1.49	32.54
16	1.22, 1.87	28.37
17	1.85	46.96
18	0.87	17.67
19	10.17	208.02
20	------	77.53
21	3.43, 3.83	75.94
22	2.33, 2.47	39.78
23	5.75	127.48
24	6.18	136.56
25	------	143.41
26	4.85	115.04
27	1.82	18.98
28	1.07	26.90
29	0.80	16.20
30	0.89	16.50
	Ara	
1'	4.45	105.08
2'	3.84	75.22
3'	3.80	81.80
4'	3.94	68.84
5'	3.47, 3.82	64.82
	Rha	
1''	5.18	102.00
2''	3.90	72.13
3''	3.69	72.14
4''	3.38	73.74
5''	3.84	70.24
6''	1.21	17.96
	Xyl	
1'''	4.44	105.08
2'''	3.29	74.62
3'''	3.33	77.41
4'''	3.51	70.96
5'''	3.22, 3.88	66.72
	Glc	
1''''	4.26	104.94
2''''	3.22	75.35
3''''	3.36	77.99
4''''	3.27	71.70
5''''	3.27	77.99
6''''	3.65, 3.87	62.77

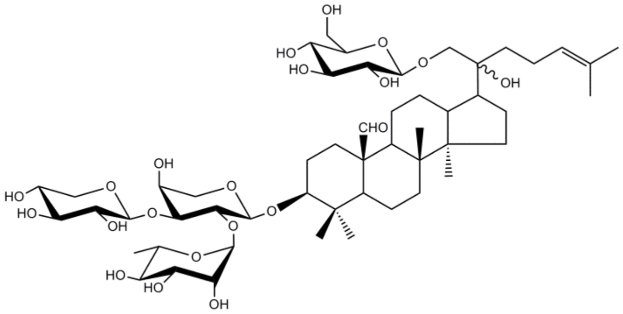

S4a. Structure of compound 10.

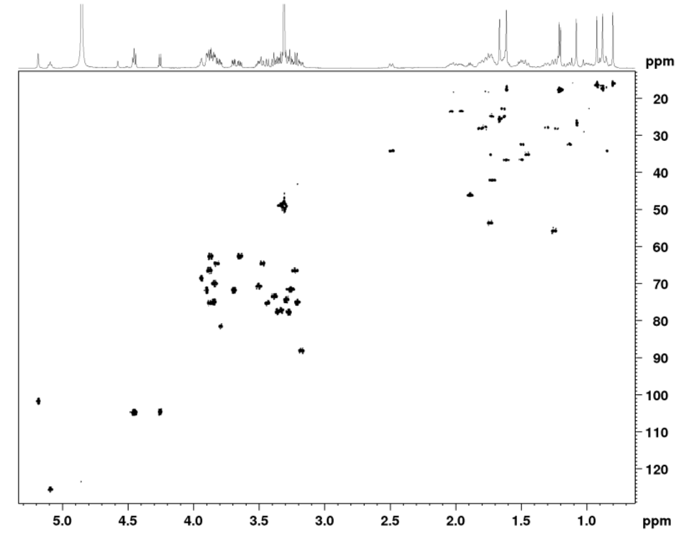

S4b. HSQC of compound 10.

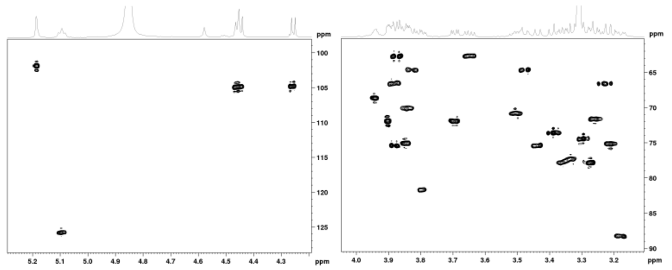

S4c. Selected region of HSQC showing anomeric and ringproton of the glycoside moiety of compound 10.

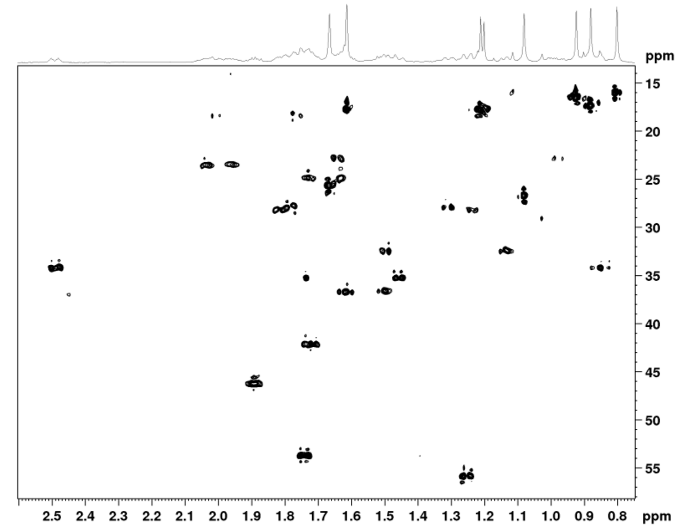

S4d. Selected region of HSQC showing the aglycone moiety of compound 10.

S4e. Chemical shifts of compound 10.

Pos.	10	
	δH	δC
1	0.85, 2.50	34.28
2	1.30, 1.78	27.95
3	3.18	88.25
4	------	40.79
5	1.25	55.89
6	1.76, 2.01	18.37
7	1.46, 1.74	35.31
8	------	41.84
9	1.74	53.77
10	------	53.96
11	0.97, 1.64	22.92
12	1.63, 1.73	24.95
13	1.72	42.20
14	------	51.04
15	1.14, 1.49	32.53
16	1.25, 1.82	28.33
17	1.89	46.29
18	0.88	17.39
19	10.18	207.91
20	------	77.28
21	3.44, 3.88	75.43
22	1.50, 1.62	36.70
23	1.96, 2.03	23.60
24	5.09	125.73
25	------	132.05
26	1.67	25.73
27	1.61	17.82
28	1.08	26.76
29	0.81	16.06
30	0.92	16.65
	Ara	
1'	4.46	104.94
2'	3.85	75.07
3'	3.8	81.66
4'	3.94	68.77
5'	3.47, 3.83	64.67
	Rha	
1''	5.18	101.86
2''	3.90	72.00
3''	3.69	72.00
4''	3.39	73.60
5''	3.84	70.10
6''	1.21	17.81
	Xyl	
1'''	4.44	104.80
2'''	3.29	74.48
3'''	3.33	77.26
4'''	3.51	70.82
5'''	3.23, 3.88	66.65
	Glc	
1''''	4.26	104.79
2''''	3.21	75.21
3''''	3.36	77.70
4''''	3.26	71.70
5''''	3.27	77.85
6''''	3.65, 3.87	62.77

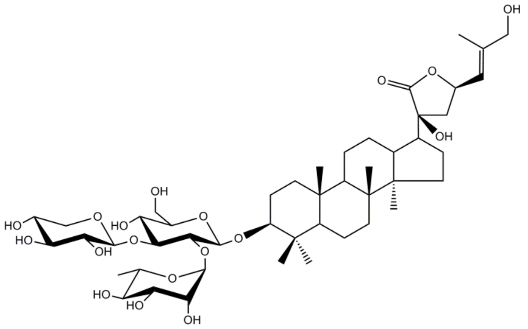

S5a. Structure of compound 12.

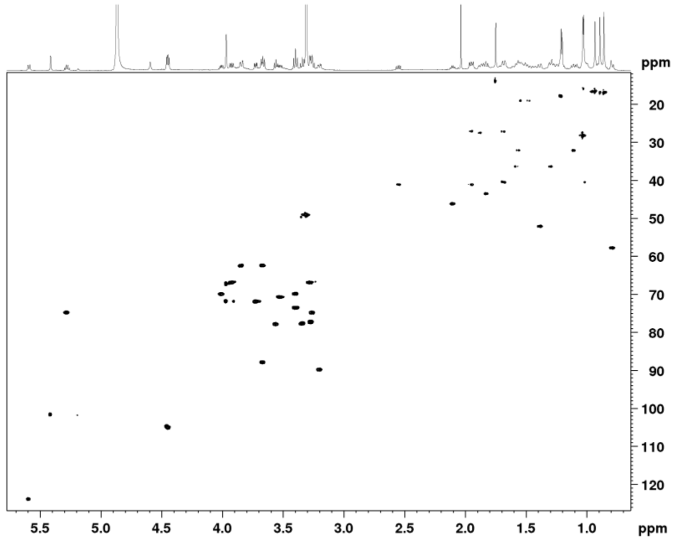

S5b. HSQC of compound 12.

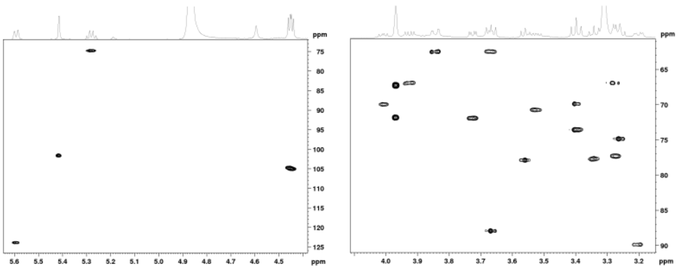

S5c. Selected region of HSQC showing anomeric and ringproton of the glycoside moiety of compound 12.

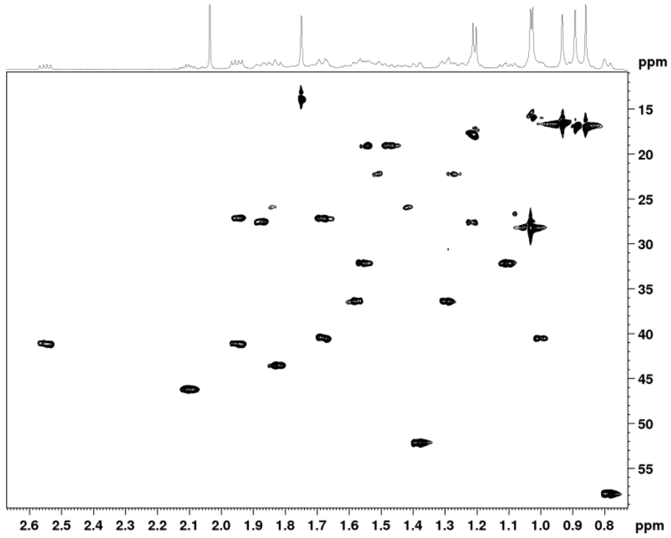

S5d. Selected region of HSQC showing the aglycone moiety of compound 12.

S5e. Chemical shifts of compound 12.

Pos.	12	
	δH	δC
1	1.00, 1.68	40.52
2	1.69, 1.94	27.19
3	3.21	89.85
4	------	40.46
5	0.79	57.78
6	1.48, 1.54	19.13
7	1.30, 1.57	36.42
8	------	41.61
9	1.39	52.23
10	------	37.88
11	1.28, 1.51	22.28
12	1.22, 1.87	27.63
13	1.83	43.59
14	------	51.47
15	1.10, 1.57	32.17
16	1.43, 1.85	26.02
17	2.11	46.23
18	1.03	16.94
19	0.89	15.92
20	------	79.38
21	------	180.43
22	1.95, 2.55	41.10
23	5.29	74.85
24	5.60	123.96
25	------	142.68
26	3.98	67.31
27	1.75	14.00
28	1.03	28.21
29	0.86	17.09
30	0.93	16.80
	Glc	
1'	4.44	105.08
2'	3.56	77.85
3'	3.67	87.95
4'	3.40	69.94
5'	3.27	77.26
6'	3.67, 3.85	62.47
	Rha	
1''	5.42	101.70
2''	3.98	71.84
3''	3.73	71.99
4''	3.40	73.59
5''	4.01	69.94
6''	1.21	17.88
	Xyl	
1'''	4.46	104.79
2'''	3.26	74.92
3'''	3.34	77.70
4'''	3.53	70.81
5'''	3.28, 3.93	67.01

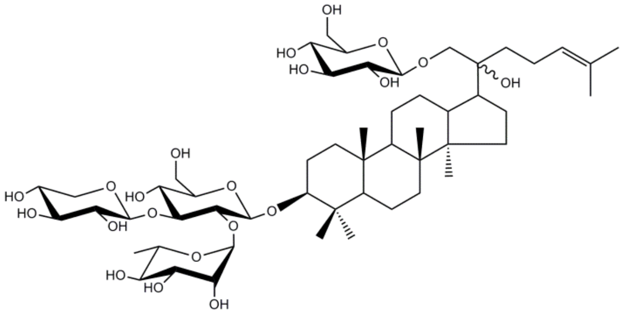

S6a. Structure of compound 15.

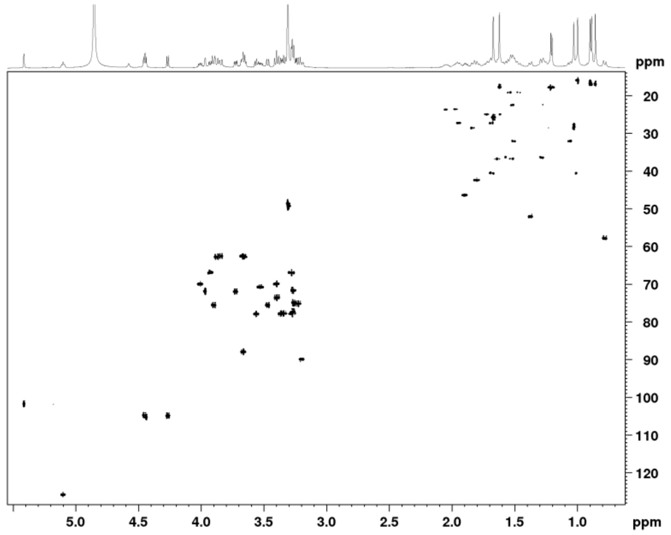

S6b. HSQC of compound 15.

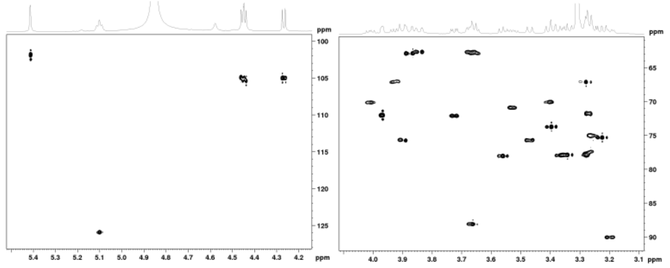

S6c. Selected region of HSQC showing anomeric and ringproton of the glycoside moiety of compound 15.

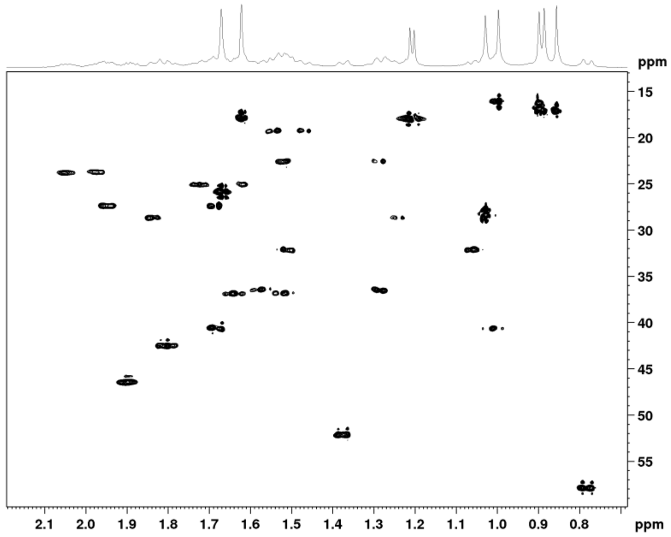

S6d. Selected region of HSQC showing the aglycone moiety of compound 15.

S6e. Chemical shifts of compound 15.

Pos.	15	
	δH	δC
1	1.01, 1.68	40.64
2	1.71, 1.95	27.41
3	3.20	90.00
4	------	40.39
5	0.78	57.95
6	1.46, 1.54	19.28
7	1.28, 1.57	36.55
8	------	41.88
9	1.38	52.22
10	------	37.81
11	1.28, 1.52	22.57
12	1.62, 1.72	25.13
13	1.80	42.55
14	------	51.30
15	1.06, 1.51	32.16
16	1.24, 1.83	28.65
17	1.90	46.51
18	0.99	16.06
19	0.89	17.16
20	------	78.02
21	3.47, 3.90	75.65
22	1.51, 1.64	36.85
23	1.97, 2.05	23.82
24	5.10	126.01
25	------	132.11
26	1.68	25.87
27	1.62	17.96
28	1.03	28.36
29	0.85	17.08
30	0.90	16.94
	Glc	
1'	4.44	105.36
2'	3.56	78.00
3'	3.66	88.10
4'	3.40	70.08
5'	3.26	77.41
6'	3.68, 3.84	62.67
	Rha	
1''	5.42	101.86
2''	3.97	71.99
3''	3.73	72.14
4''	3.40	73.75
5''	4.01	70.09
6''	1.21	17.96
	Xyl	
1'''	4.45	104.93
2'''	3.26	75.06
3'''	3.33	77.85
4'''	3.53	70.97
5'''	3.28, 3.93	67.14
	Glc	
1''''	4.26	104.93
2''''	3.22	75.35
3''''	3.37	77.98
4''''	3.27	71.84
5''''	3.28	77.85
6''''	3.65, 3.87	62.91

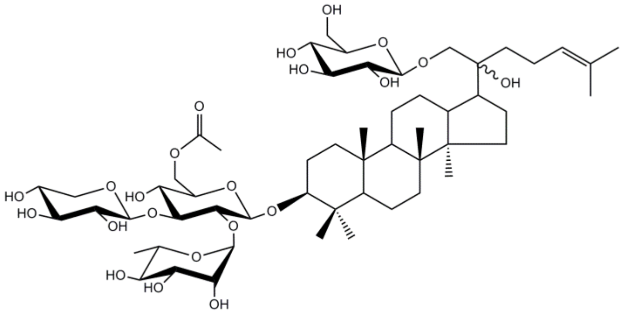

S7a. Structure of compound 17.

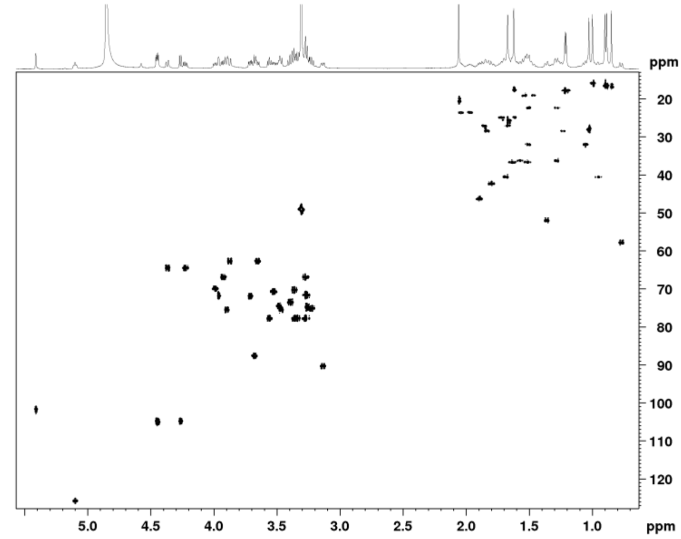

S7b. HSQC of compound 17.

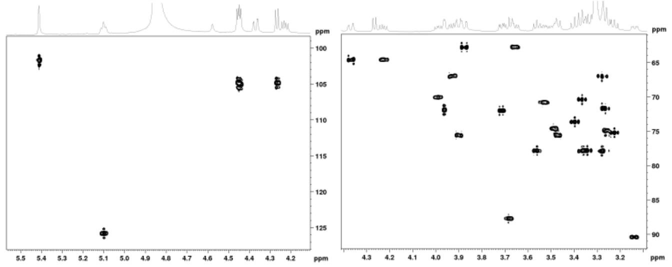

S7c. Selected region of HSQC showing anomeric and ringproton of the glycoside moiety of compound 17.

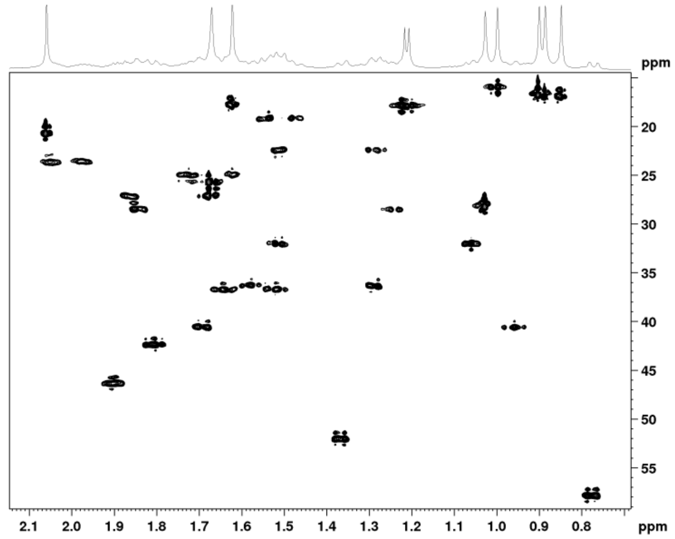

S7d. Selected region of HSQC showing the aglycone moiety of compound 17.

S7e. Chemical shifts of compound 17.

Pos.	17	
	δH	δC
1	0.96, 1.69	40.66
2	1.66, 1.86	27.19
3	3.14	90.30
4	------	40.39
5	0.78	57.94
6	1.47, 1.54	19.15
7	1.28, 1.57	36.42
8	------	41.89
9	1.36	52.08
10	------	37.81
11	1.28, 1.51	22.37
12	1.62, 1.71	24.99
13	1.80	42.42
14	------	51.10
15	1.06, 1.52	32.02
16	1.24, 1.84	28.51
17	1.90	46.37
18	0.99	15.92
19	0.89	16.94
20	------	77.99
21	3.47, 3.90	75.51
22	1.52, 1.64	36.71
23	1.97, 2.05	23.68
24	5.10	125.80
25	------	132.09
26	1.68	25.72
27	1.62	17.83
28	1.03	28.22
29	0.85	16.95
30	0.90	16.79
	Glc	
1'	4.45	104.94
2'	3.56	77.85
3'	3.69	87.67
4'	3.37	70.39
5'	3.49	74.49
6'	4.22, 4.37	64.53
-O***C***OCH3	------	172.51
-OCO***CH***3	2.06	20.74
	Rha	
1''	5.42	101.72
2''	3.97	71.85
3''	3.72	71.99
4''	3.40	73.60
5''	3.99	70.09
6''	1.22	17.82
	Xyl	
1'''	4.46	104.79
2'''	3.26	74.92
3'''	3.33	77.70
4'''	3.53	70.81
5'''	3.28, 3.93	67.04
	Glc	
1''''	4.27	104.79
2''''	3.22	75.21
3''''	3.37	77.84
4''''	3.27	71.70
5''''	3.28	77.85
6''''	3.65, 3.88	62.77

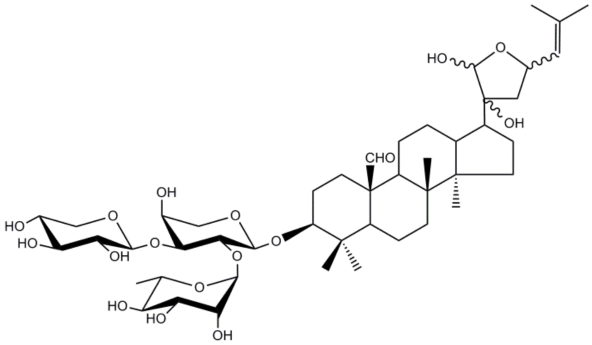

S8a. Structure of compound 18.

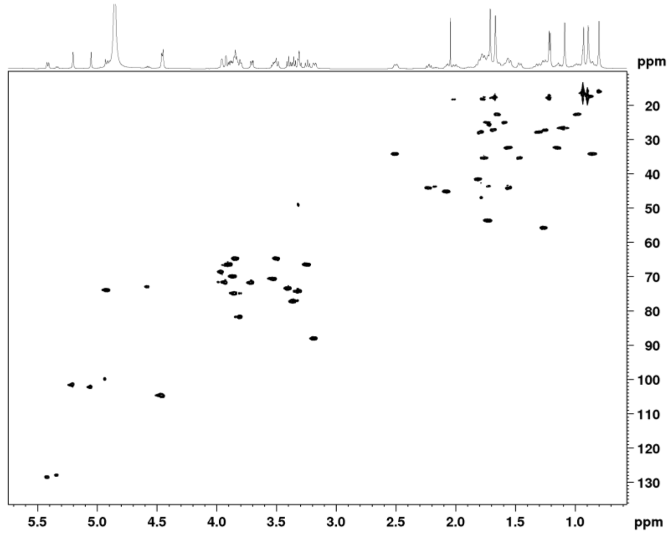

S8b. HSQC of compound 18.

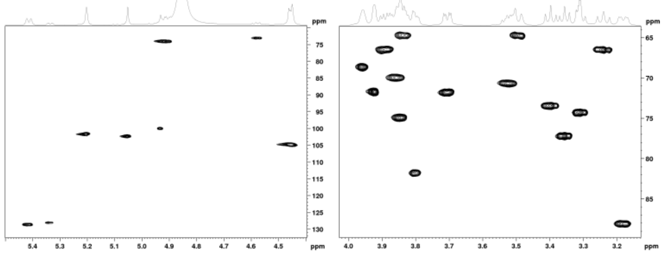

S8c. Selected region of HSQC showing anomeric and ringproton of the glycoside moiety of compound 18.

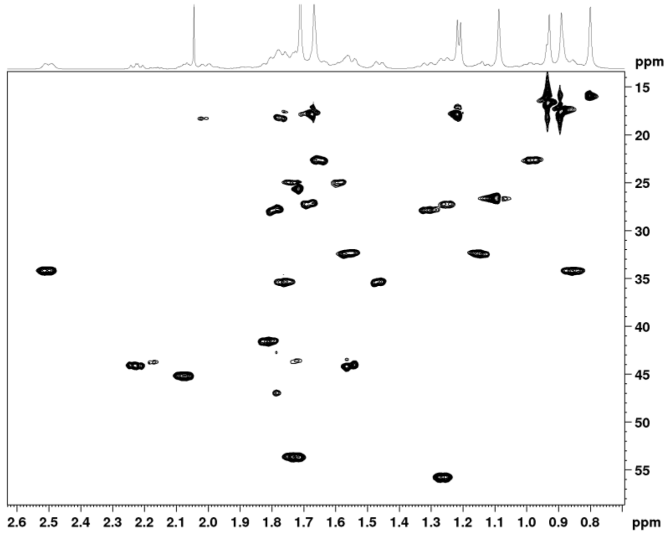

S8d. Selected region of HSQC showing the aglycone moiety of compound 18.

S8e. Chemical shifts of compound 18.

Pos.	18 (major)		18 (minior)	
	δH	δC	δH	δC
1	0.85, 2.50	34.28	0.85, 2.50	34.28
2	1.31, 1.78	27.99	1.31, 1.78	27.99
3	3.18	88.25	3.18	88.25
4	------	40.81	------	40.81
5	1.25	55.87	1.25	55.87
6	1.77, 2.02	18.27	1.77, 2.02	18.27
7	1.46, 1.75	35.38	1.46, 1.75	35.38
8	------	41.30	------	41.30
9	1.72	53.83	1.72	53.83
10	------	53.87	------	53.87
11	0.97, 1.64	22.74	0.97, 1.64	22.74
12	1.57, 1.73	25.06	1.57, 1.73	25.06
13	1.81	41.69	1.79	42.71
14	------	50.33	------	50.33
15	1.14, 1,55	32.45	1.14, 1,55	32.45
16	1.24, 1.68	27.29	1.24, 1.68	27.29
17	2.07	45.35	1.78	46.95
18	0.89	17.60	0.89	17.54
19	10.18	208.05	10.18	208.05
20	------	85.32	------	82.80
21	5.04	102.45	4.94	99.96
22	1.55, 2.22	44.16	1.72, 2.17	43.73
23	4.91	74.16	4.58	73.02
24	5.41	128.81	5.34	128.06
25	------	134.86	------	135.55
26	1.71	25.70	1.71	25.70
27	1.67	17.82	1.66	18.40
28	1.08	26.75	1.08	26.75
29	0.80	16.06	0.80	16.06
30	0.92	16.58	0.93	16.79
	Ara		*Ara*	
1'	4.46	104.94	4.46	104.94
2'	3.85	75.06	3.85	75.06
3'	3.80	81.74	3.80	81.74
4'	3.94	68.70	3.94	68.70
5'	3.48, 3.83	64.75	3.48, 3.83	64.75
	Rha		*Rha*	
1''	5.19	101.86	5.19	101.86
2''	3.91	71.99	3.91	71.99
3''	3.69	71.99	3.69	71.99
4''	3.39	73.60	3.39	73.60
5''	3.84	70.09	3.84	70.09
6''	1.21	17.82	1.21	17.82
	Xyl		*Xyl*	
1'''	4.45	104.86	4.45	104.86
2'''	3.29	74.48	3.29	74.48
3'''	3.33	77.34	3.33	77.34
4'''	3.51	70.85	3.51	70.85
5'''	3.23, 3.88	66.65	3.23, 3.88	66.65

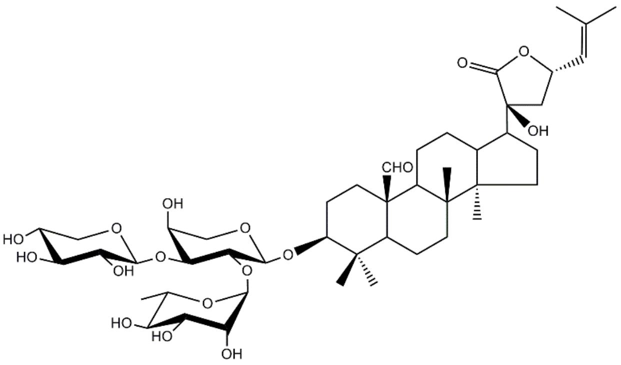

S9a. Structure of compound 19.

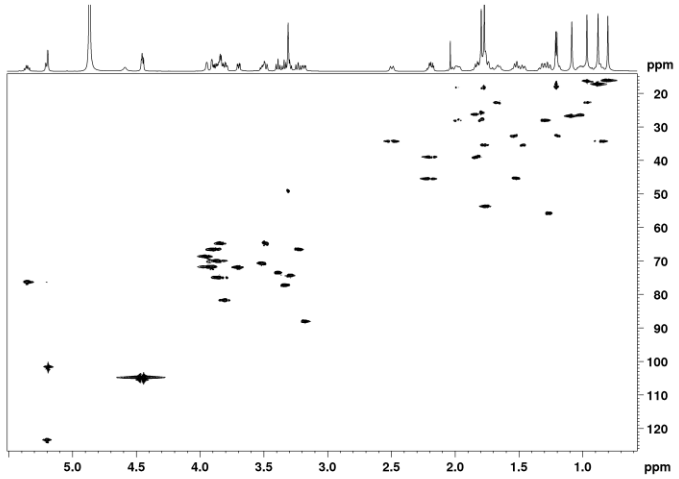

S9b. HSQC of compound 19.

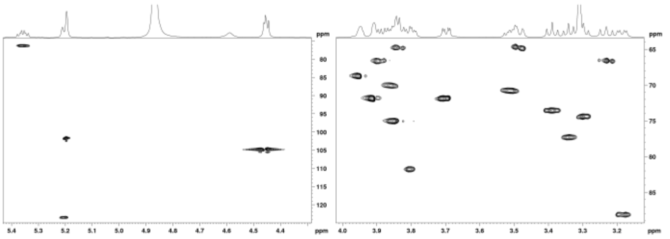

S9c. Selected region of HSQC showing anomeric and ringproton of the glycoside moiety of compound 19.

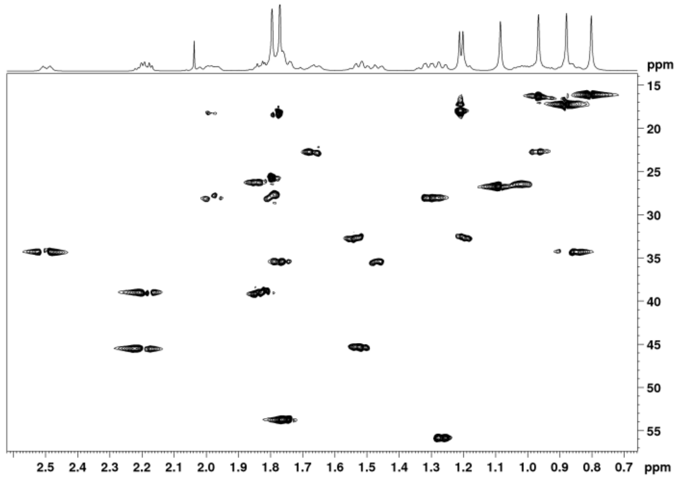

S9d. Selected region of HSQC showing the aglycone moiety of compound 19.

S9e. Chemical shifts of compound 19.

Pos.	19	
	δH	δC
1	0.85, 2.50	34.43
2	1.28, 1.96	28.06
3	3.19	88.32
4	------	40.72
5	1.26	55.96
6	1.78, 2.01	18.41
7	1.46, 1.76	35.46
8	------	41.20
9	1.75	53.76
10	------	53.87
11	0.97, 1.66	22.87
12	1.31, 1.78	28.29
13	1.52	45.35
14	------	50.80
15	1.20, 1.53	32.68
16	1.02, 1.84	26.39
17	2.19	45.49
18	0.88	17.23
19	10.18	207.79
20	------	81.77
21	------	179.29
22	1.82, 2.18	39.12
23	5.36	76.46
24	5.20	123.74
25	------	141.18
26	1.79	25.79
27	1.77	18.25
28	1.08	26.76
29	0.81	16.13
30	0.97	16.36
	Ara	
1'	4.46	104.85
2'	3.85	75.14
3'	3.80	81.72
4'	3.94	68.84
5'	3.47, 3.83	64.74
	Rha	
1''	5.19	101.92
2''	3.90	71.99
3''	3.69	72.06
4''	3.39	73.67
5''	3.84	70.16
6''	1.21	17.89
	Xyl	
1'''	4.44	104.93
2'''	3.29	74.55
3'''	3.33	77.41
4'''	3.51	70.89
5'''	3.22, 3.88	66.79

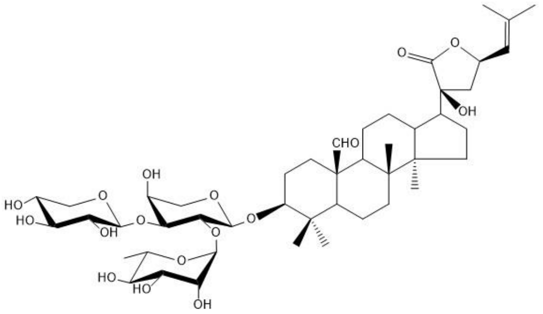

S10a. Structure of compound 20A.

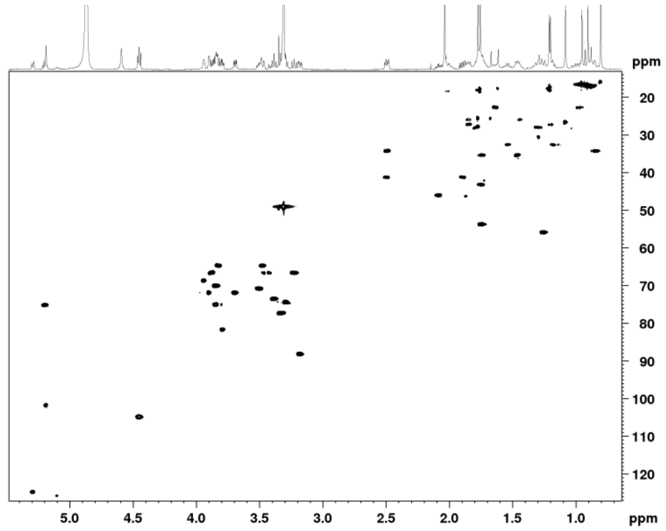

S10b. HSQC of compound 20A.

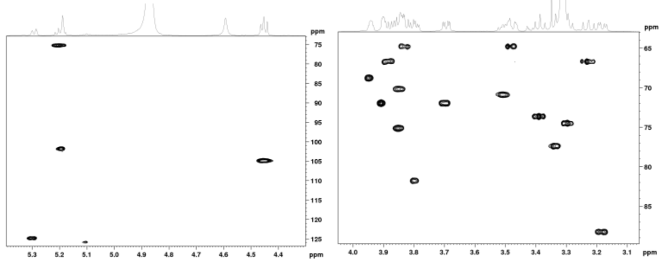

S10c. Selected region of HSQC showing anomeric and ringproton of the glycoside moiety of compound 20A.

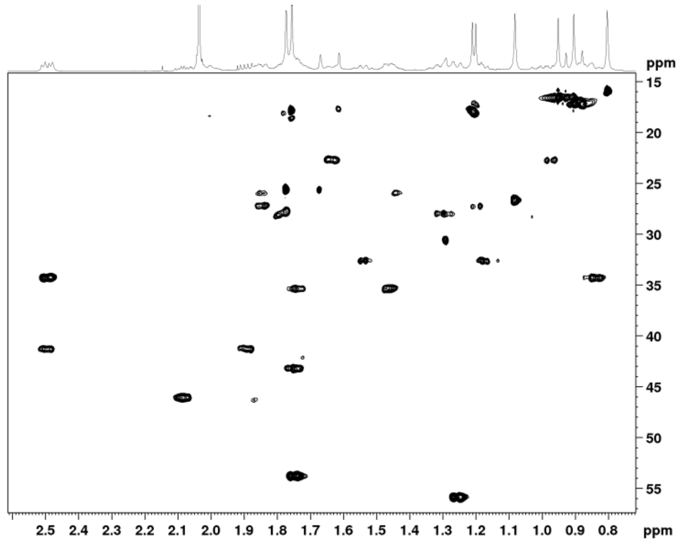

S10d. Selected region of HSQC showing the aglycone moiety of compound 20A.

S10e. Chemical shifts of compound 20A.

Pos.	20A	
	δH	δC
1	0.85, 2.50	34.37
2	1.30, 1.79	28.06
3	3.19	88.24
4	------	40.71
5	1.26	55.88
6	1.79, 2.02	18.40
7	1.46, 1.75	35.39
8	------	41.42
9	1.75	53.84
10	------	53.85
11	0.96, 1.64	22.72
12	1.44, 1.85	26.01
13	1.75	43.22
14	------	51.27
15	1.18, 1.53	32.61
16	1.19, 1.84	27.19
17	2.09	46.08
18	0.91	17.09
19	10.19	207.23
20	------	79.28
21	------	179.88
22	1.88, 2.50	41.25
23	5.20	75.21
24	5.30	124.84
25	------	140.34
26	1.78	25.73
27	1.76	17.97
28	1.09	26.75
29	0.81	16.07
30	0.96	16.65
	Ara	
1'	4.46	104.93
2'	3.85	75.06
3'	3.80	81.79
4'	3.94	68.76
5'	3.48, 3.83	64.74
	Rha	
1''	5.19	101.85
2''	3.91	71.98
3''	3.69	71.99
4''	3.39	73.60
5''	3.85	70.08
6''	1.21	17.97
	Xyl	
1'''	4.44	104.93
2'''	3.29	74.47
3'''	3.33	77.40
4'''	3.51	70.82
5'''	3.23, 3.88	66.64

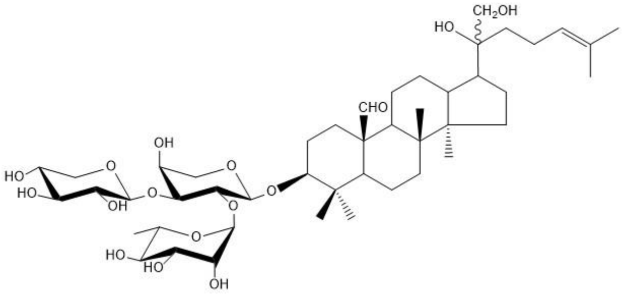

S11a. Structure of compound 20B.

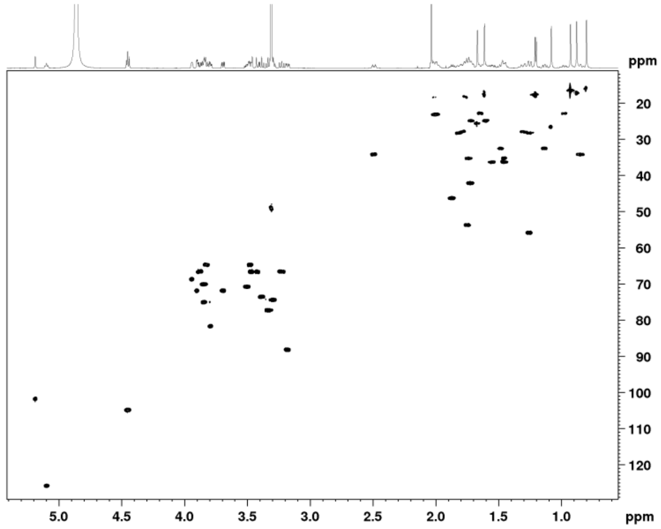

S11b. HSQC of compound 20B.

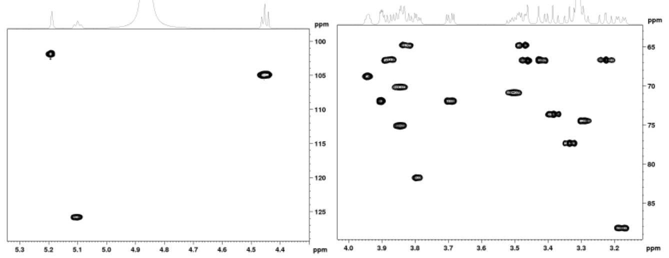

S11c. Selected region of HSQC showing anomeric and ringproton of the glycoside moiety of compound 20B.

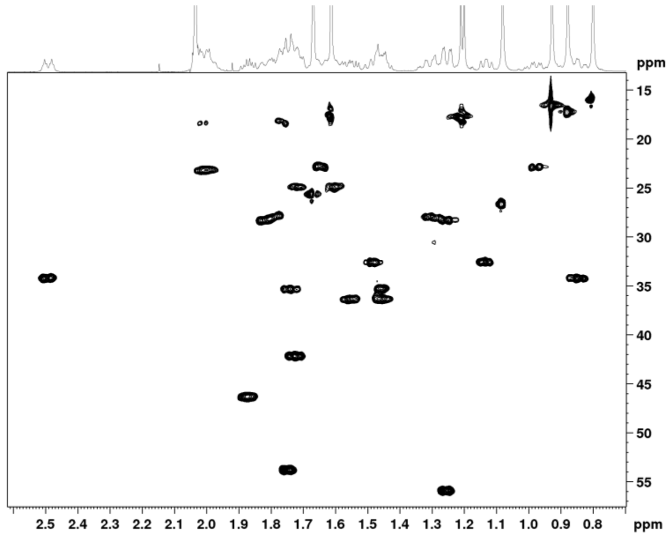

S11d. Selected region of HSQC showing the aglycone moiety of compound 20B.

S11e. Chemical shifts of compound 20B.

Pos.	20B	
	δH	δC
1	0.85,2.49	34.22
2	1.31, 1.77	27.98
3	3.18	88.24
4	------	40.70
5	1.26	55.88
6	1.77, 2.01	18.39
7	1.46, 1.73	35.37
8	------	41.17
9	1.75	53.83
10	------	53.84
11	0.98, 1.64	22.94
12	1.60, 1.72	24.99
13	1.73	42.12
14	------	51.02
15	1.13, 1.48	32.61
16	1.24, 1.82	28.36
17	1.87	46.36
18	0.88	17.38
19	10.18	207.88
20	------	77.53
21	3.42, 3.47	66.72
22	1.45, 1.55	36.41
23	1.98, 2.01	23.24
24	5.10	125.86
25	------	131.91
26	1.67	25.72
27	1.62	17.67
28	1.09	26.74
29	0.80	16.07
30	0.93	16.65
	Ara	
1'	4.46	104.95
2'	3.85	75.06
3'	3.80	81.80
4'	3.94	68.76
5'	3.48, 3.82	64.75
	Rha	
1''	5.19	101.85
2''	3.90	71.91
3''	3.70	71.90
4''	3.39	73.60
5''	3.85	70.09
6''	1.21	17.96
	Xyl	
1'''	4.45	104.95
2'''	3.29	74.47
3'''	3.34	77.32
4'''	3.51	70.82
5'''	3.23, 3.88	66.64

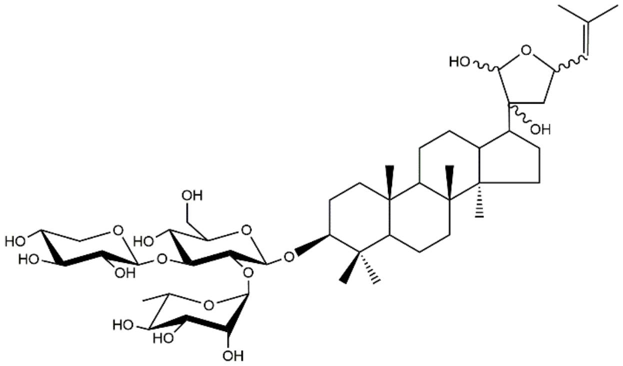

S12a. Structure of compound 21.

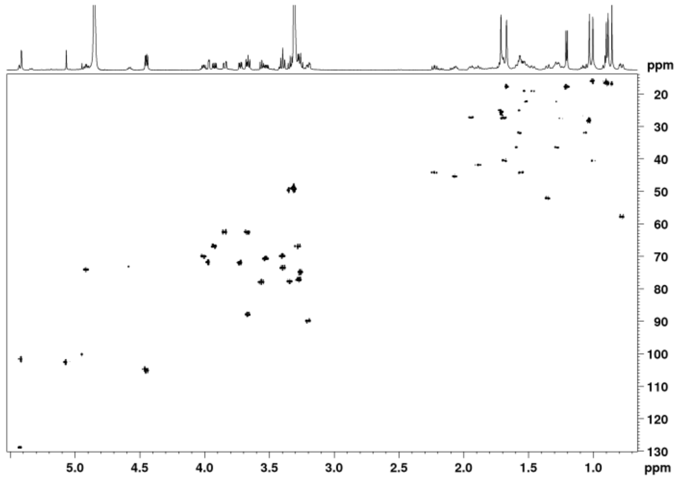

S12b. HSQC of compound 21.

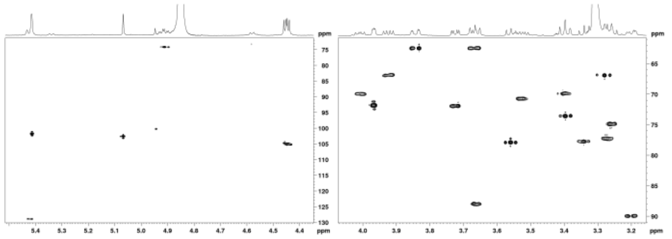

S12c. Selected region of HSQC showing anomeric and ringproton of the glycoside moiety of compound 21.

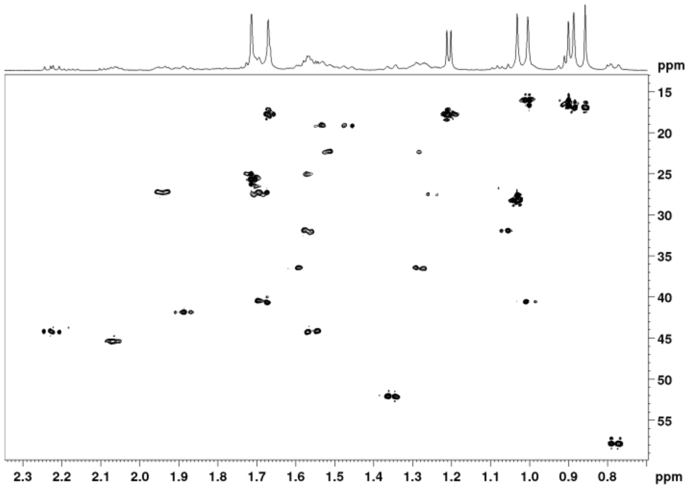

S12d. Selected region of HSQC showing the aglycone moiety of compound 21.

S12e. Chemical shifts of compound 21.

Pos.	21 (major)		21 (minor)	
	δH	δC	δH	δC
1	1.01, 1.68	40.65	1.01, 1.68	40.65
2	1.68, 1.94	27.33	1.68, 1.94	27.33
3	3.20	89.89	3.20	89.89
4	------	40.38	------	40.38
5	0.78	57.80	0.78	57.80
6	1.46, 1.54	19.14	1.46, 1.54	19.14
7	1.60, 1.28	36.41	1.60, 1.28	36.41
8	------	41.71	------	41.71
9	1.35	52.08	1.35	52.08
10	------	37.81	------	37.81
11	1.28, 1.51	22.36	1.28, 1.51	22.36
12	1.26, 1.70	27.63	1.26, 1.70	27.63
13	1.89	41.83	1.85	43.06
14	------	51.52	------	51.52
15	1.06, 1.57	32.02	1.06, 1.57	32.02
16	1.57, 1.73	25.00	1.57, 1.73	25.00
17	2.07	45.41	1.79	47.23
18	1.00	16.06	1.00	16.06
19	0.89	16.94	0.89	16.94
20	------	85.61	------	83.03
21	5.07	102.60	4.95	100.25
22	1.56, 2.24	44.17	1.71, 2.18	43.80
23	4.91	74.18	4.58	73.16
24	5.42	128.84	5.34	128.22
25	------	134.67	------	135.78
26	1.71	25.65	1.71	25.65
27	1.68	17.75	1.67	17.97
28	1.03	28.21	1.03	28.21
29	0.85	16.94	0.85	16.94
30	0.90	16.65	0.91	17.07
	Glc		*Glc*	
1'	4.45	105.15	4.45	105.15
2'	3.56	77.85	3.56	77.85
3'	3.67	87.95	3.67	87.95
4'	3.40	69.94	3.40	69.94
5'	3.27	77.26	3.27	77.26
6'	3.67, 3.85	62.48	3.67, 3.85	62.48
	Rha		*Rha*	
1''	5.42	101.72	5.42	101.72
2''	3.97	71.84	3.97	71.84
3''	3.73	71.99	3.73	71.99
4''	3.40	73.60	3.40	73.60
5''	4.01	69.94	4.01	69.94
6''	1.21	17.82	1.21	17.82
	Xyl		*Xyl*	
1'''	4.46	104.78	4.46	104.78
2'''	3.26	74.92	3.26	74.92
3'''	3.34	77.71	3.34	77.71
4'''	3.53	70.82	3.53	70.82
5'''	3.28, 3.93	66.87	3.28, 3.93	66.87

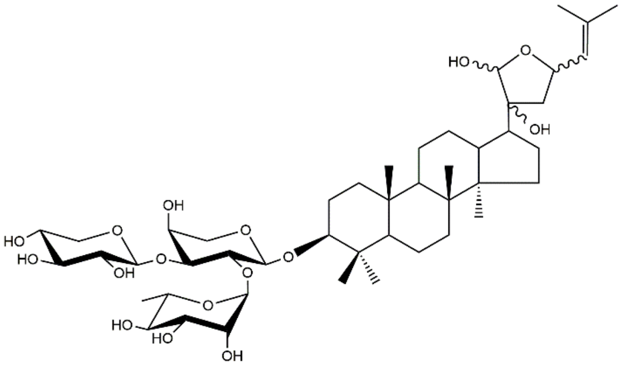

S13a. Structure of compound 22.

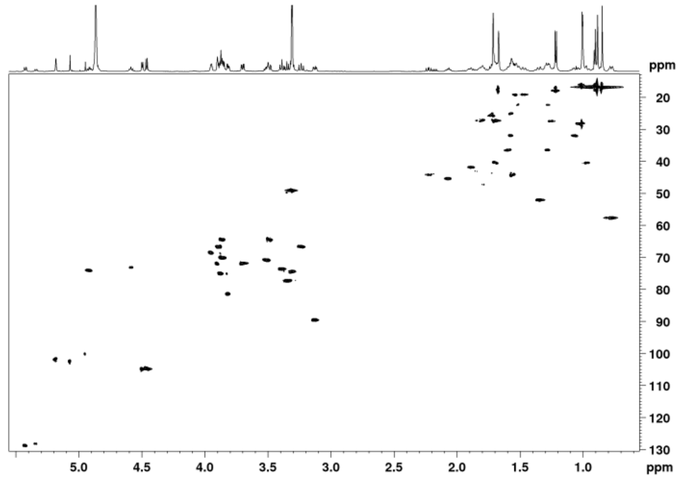

S13b. HSQC of compound 22.

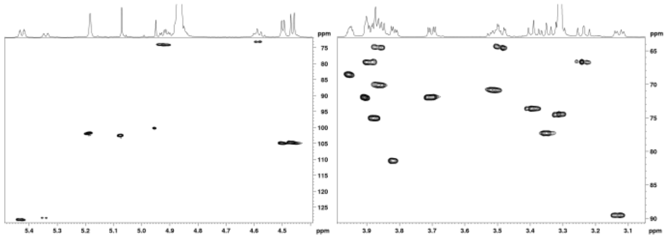

S13c. Selected region of HSQC showing anomeric and ringproton of the glycoside moiety of compound 22.

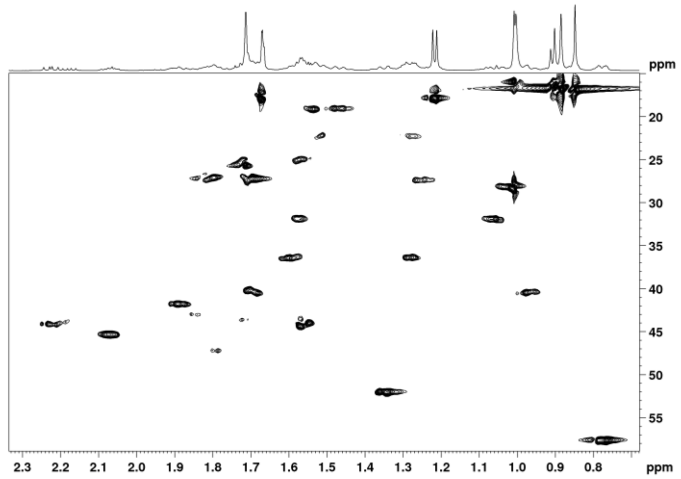

S13d. Selected region of HSQC showing the aglycone moiety of compound 22.

S13e. Chemical shifts of compound 22.

Pos.	22 (major)		22 (minor)	
	δH	δC	δH	δC
1	0.97, 1.69	40.50	0.97, 1.69	40.50
2	1.69, 1.80	27.20	1.69, 1.80	27.20
3	3.13	89.56	3.13	89.56
4	------	40.48	------	40.48
5	0.76	57.64	0.76	57.64
6	1.47, 1.53	19.14	1.47, 1.53	19.14
7	1.28, 1.60	36.41	1.28, 1.60	36.41
8	------	41.57	------	41.57
9	1.34	52.07	1.34	52.07
10	------	37.90	------	37.90
11	1.28, 1.51	22.36	1.28, 1.51	22.36
12	1.25, 1.71	27.48	1.25, 1.71	27.48
13	1.89	41.83	1.84	43.15
14	------	50.79	------	50.79
15	1.06, 1.57	32.02	1.06, 1.57	32.02
16	1.57, 1.71	24.99	1.57, 1.71	24.99
17	2.07	45.48	1.79	47.25
18	1.01	15.99	1.01	15.99
19	0.89	16.94	0.89	16.94
20	------	85.49	------	82.91
21	5.07	102.50	4.95	100.24
22	1.56, 2.23	44.17	1.71, 2.18	43.73
23	4.92	74.04	4.58	73.15
24	5.43	128.79	5.34	128.21
25	------	134.71	------	135.67
26	1.71	25.73	1.71	25.73
27	1.68	17.90	1.67	17.96
28	1.01	28.43	1.01	28.43
29	0.85	16.93	0.85	16.93
30	0.90	16.51	0.91	17.23
	Ara		*Ara*	
1'	4.50	104.93	4.50	104.93
2'	3.87	75.06	3.87	75.06
3'	3.82	81.36	3.82	81.36
4'	3.95	68.47	3.95	68.47
5'	3.49, 3.87	64.37	3.49, 3.87	64.37
	Rha		*Rha*	
1''	5.18	101.86	5.18	101.86
2''	3.90	71.99	3.90	71.99
3''	3.70	71.99	3.70	71.99
4''	3.39	73.60	3.39	73.60
5''	3.86	70.15	3.86	70.15
6''	1.21	17.97	1.21	17.97
	Xyl		*Xyl*	
1'''	4.46	104.79	4.46	104.79
2'''	3.31	74.48	3.31	74.48
3'''	3.35	77.26	3.35	77.26
4'''	3.51	70.82	3.51	70.82
5'''	3.24, 3.89	66.63	3.24, 3.89	66.63

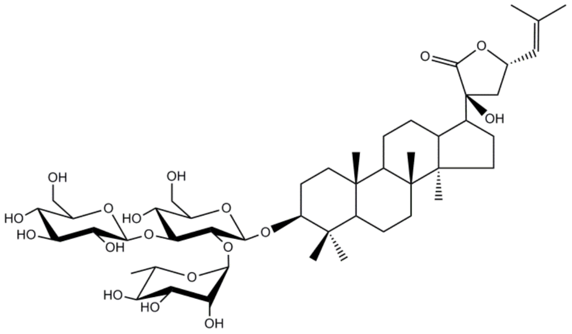

S14a. Structure of compound 23.

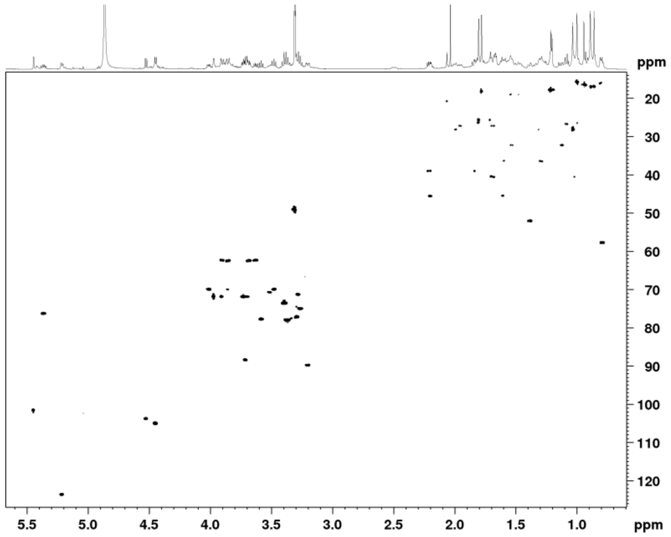

S14b. HSQC of compound 23.

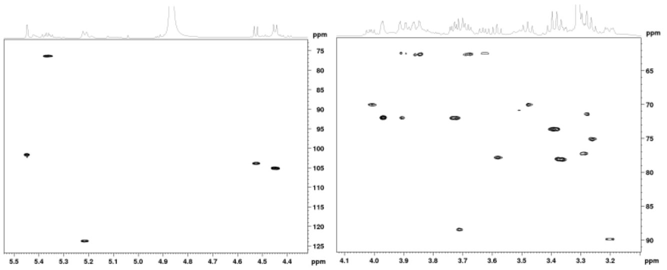

S14c. Selected region of HSQC showing anomeric and ringproton of the glycoside moiety of compound 23.

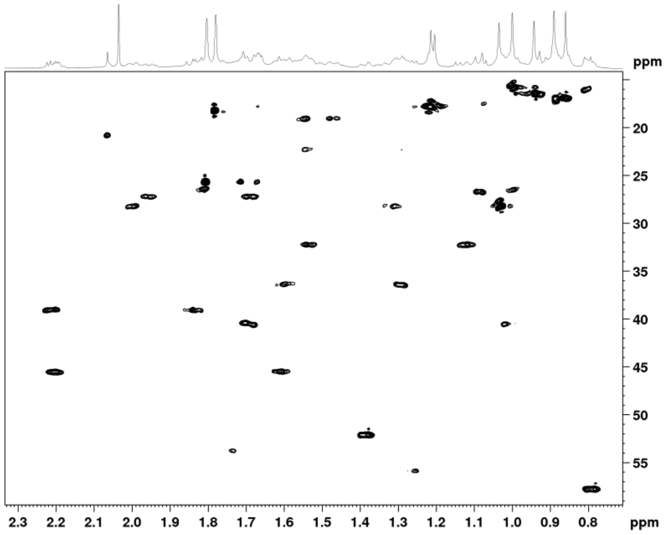

S14d. Selected region of HSQC showing the aglycone moiety of compound 23.

S14e. Chemical shifts of compound 23.

Pos.	23	
	δH	δC
1	1.02, 1.69	40.51
2	1.69, 1.96	27.18
3	3.20	89.85
4	------	40.47
5	0.80	57.79
6	1.47, 1.54	19.13
7	1.30, 1.60	36.41
8	------	41.64
9	1.38	52.08
10	------	38.11
11	1.29, 1.53	22.36
12	1.32, 1.99	28.21
13	1.61	45.49
14	------	51.01
15	1.12, 1.53	32.31
16	1.00, 1.82	26.59
17	2.20	45.64
18	0.99	15.91
19	0.89	17.09
20	------	82.03
21	------	179.33
22	1.84, 2.21	39.05
23	5.37	76.38
24	5.22	123.68
25	------	141.17
26	1.81	25.72
27	1.78	18.26
28	1.03	28.21
29	0.86	16.93
30	0.94	16.50
	Glc	
1'	4.45	105.08
2'	3.58	77.84
3'	3.72	88.39
4'	3.48	69.94
5'	3.29	77.26
6'	3.69, 3.86	62.62
	Rha	
1''	5.45	101.57
2''	3.98	71.84
3''	3.73	71.99
4''	3.40	73.60
5''	4.01	69.94
6''	1.22	17.81
	Glc	
1'''	4.53	103.83
2'''	3.26	75.07
3'''	3.37	78.05
4'''	3.48	69.94
5'''	3.29	77.26
6'''	3.63, 3.91	62.47

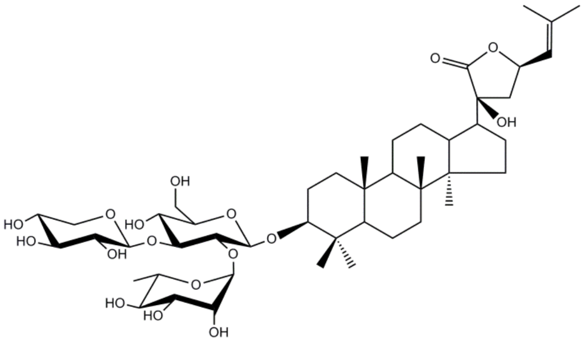

S15a. Structure of compound 25.

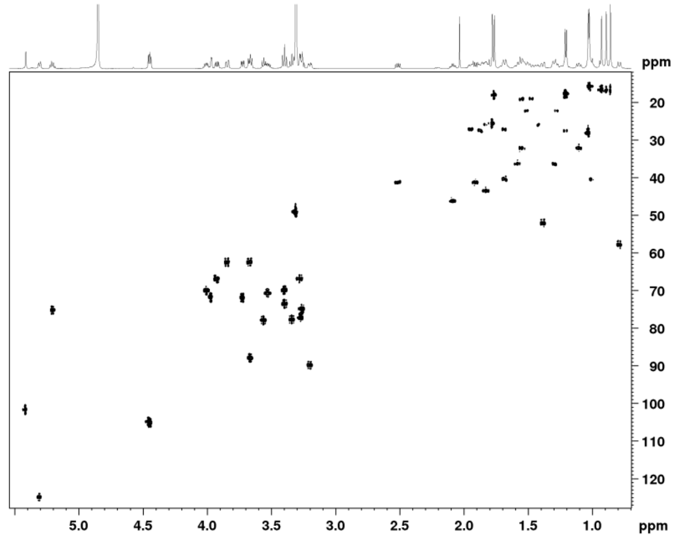

S15b. HSQC of compound 25.

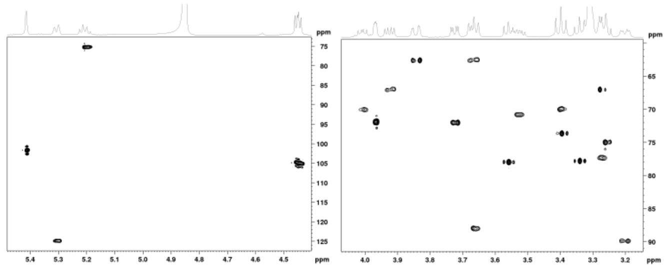

S15c. Selected region of HSQC showing anomeric and ringproton of the glycoside moiety of compound 25.

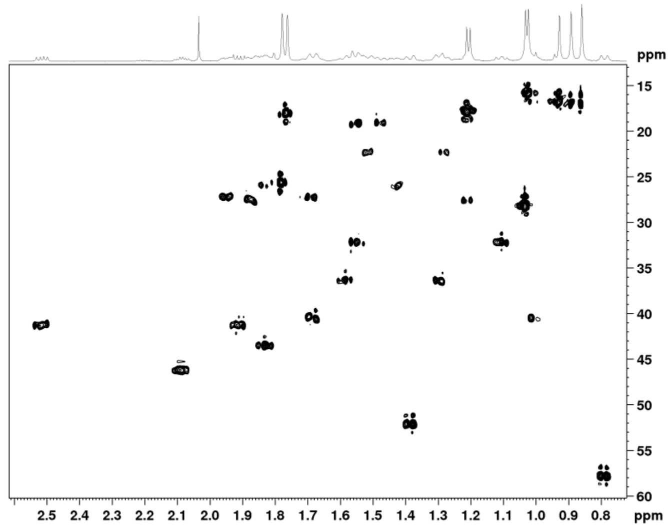

S15d. Selected region of HSQC showing the aglycone moiety of compound 25.

S15e. Chemical shifts of compound 25.

Pos.	25	
	δH	δC
1	1.01, 1.68	40.53
2	1.69, 1.95	27.27
3	3.20	89.86
4	------	40.45
5	0.79	57.93
6	1.48, 1.56	19.13
7	1.30, 1.58	36.41
8	------	41.55
9	1.39	52.21
10	------	37.86
11	1.28, 1.51	22.35
12	1.21, 1.88	27.63
13	1.83	43.59
14	------	51.59
15	1.10, 1.56	32.18
16	1.42, 1.83	26.02
17	2.09	46.22
18	1.02	15.78
19	0.89	16.95
20	------	79.44
21	------	180.71
22	1.91, 2.52	41.39
23	5.20	75.21
24	5.31	124.84
25	------	140.29
26	1.78	25.64
27	1.77	18.05
28	1.03	28.22
29	0.86	17.01
30	0.93	16.79
	Glc	
1'	4.44	105.07
2'	3.56	77.99
3'	3.67	87.95
4'	3.40	69.94
5'	3.27	77.26
6'	3.67, 3.84	62.48
	Rha	
1''	5.41	101.72
2''	3.97	71.84
3''	3.73	71.99
4''	3.40	73.60
5''	4.01	70.01
6''	1.21	17.89
	Xyl	
1'''	4.45	104.93
2'''	3.26	74.92
3'''	3.34	77.70
4'''	3.53	70.75
5'''	3.28, 3.93	67.01

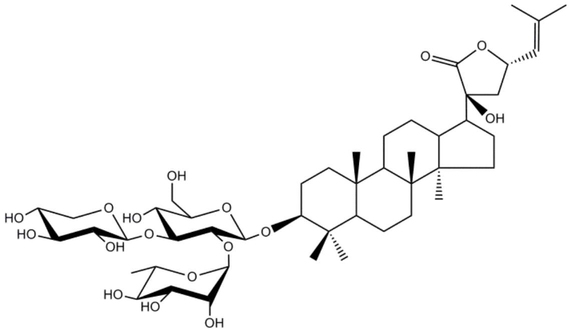

S16a. Structure of compound 26.

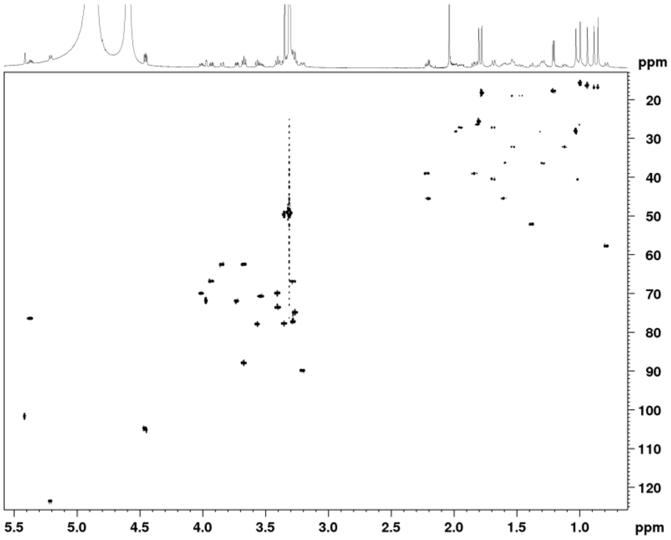

S16b. HSQC of compound 26.

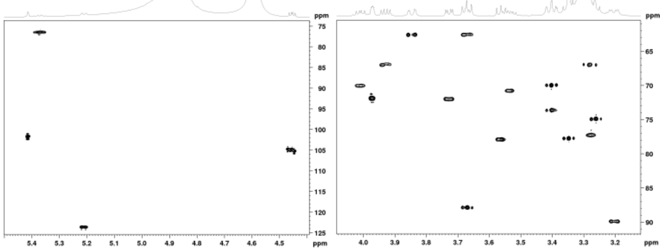

S16c. Selected region of HSQC showing anomeric and ringproton of the glycoside moiety of compound 26.

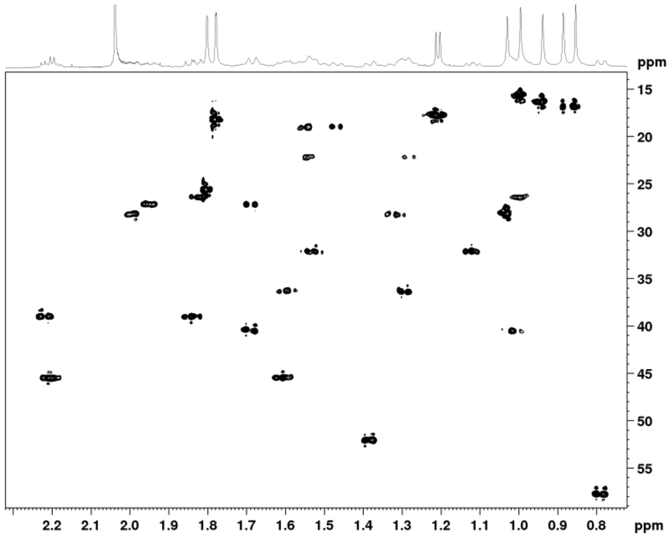

S16d. Selected region of HSQC showing the aglycone moiety of compound 26.

S16e. Chemical shifts of compound 26.

Pos.	26	
	δH	δC
1	1.02, 1.69	40.65
2	1.69, 1.95	27.27
3	3.21	90.00
4	------	40.39
5	0.79	57.79
6	1.46, 1.55	19.07
7	1.29, 1.59	36.41
8	------	41.48
9	1.39	52.08
10	------	37.81
11	1.28, 1.53	22.22
12	1.32, 1.98	28.22
13	1.61	45.49
14	------	51.08
15	1.12, 1.53	32.16
16	1.00, 1.82	26.46
17	2.21	45.64
18	0.99	15.92
19	0.89	16.94
20	------	81.74
21	------	179.45
22	1.84, 2.22	39.05
23	5.37	76.39
24	5.21	123.69
25	------	141.30
26	1.80	25.73
27	1.78	18.26
28	1.03	28.21
29	0.85	16.94
30	0.94	16.42
	Glc	
1'	4.45	105.22
2'	3.56	77.84
3'	3.67	87.95
4'	3.40	69.94
5'	3.28	77.26
6'	3.67, 3.85	62.47
	Rha	
1''	5.42	101.72
2''	3.98	71.84
3''	3.73	72.00
4''	3.40	73.60
5''	4.01	69.94
6''	1.21	17.82
	Xyl	
1'''	4.46	104.79
2'''	3.26	74.92
3'''	3.35	77.70
4'''	3.54	70.73
5'''	3.28, 3.93	66.95

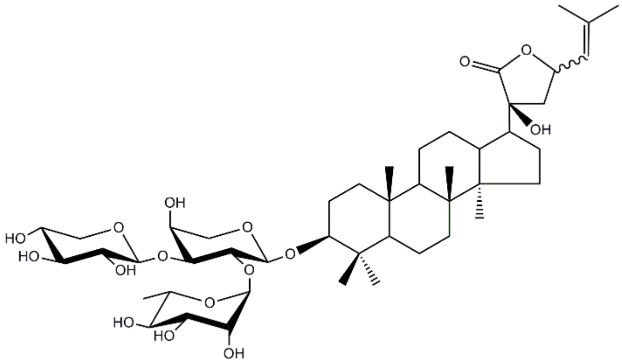

S17a. Structure of compound 27.

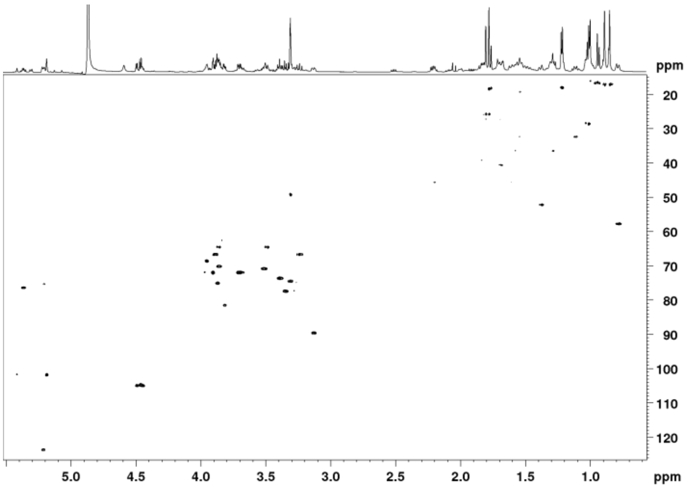

S17b. HSQC of compound 27.

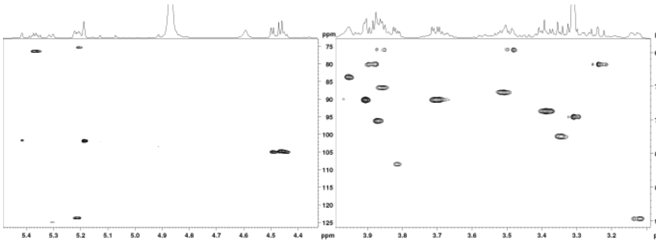

S17c. Selected region of HSQC showing anomeric and ringproton of the glycoside moiety of compound 27.

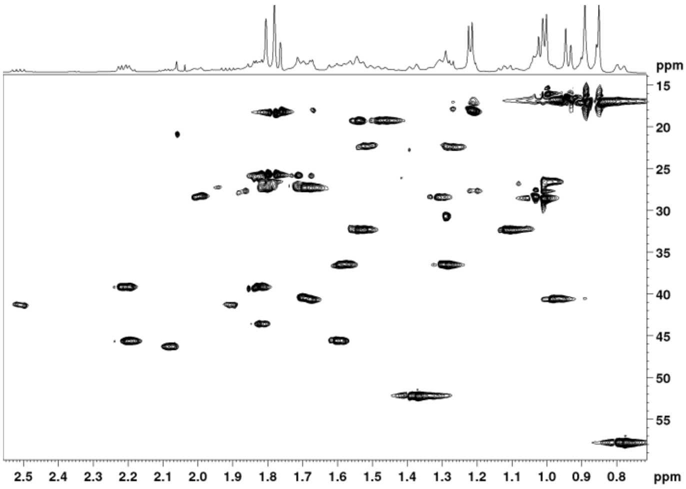

S17d. Selected region of HSQC showing the aglycone moiety of compound 27.

S17e. Chemical shifts of compound 27.

Pos.	27A		27B	
	δH	δC	δH	δC
1	0.98, 1.69	40.51	0.98, 1.69	40.51
2	1.69, 1.81	27.19	1.69, 1.81	27.19
3	3.13	89.56	3.13	89.56
4	------	40.49	------	40.49
5	0.78	57.79	0.78	57.79
6	1.47, 1.54	19.14	1.47, 1.54	19.14
7	1.28, 1.59	36.42	1.28, 1.59	36.42
8	------	41.66	------	41.66
9	1.37	52.15	1.37	52.15
10	------	37.91	------	37.91
11	1.27, 1.51	22.34	1.27, 1.51	22.34
12	1.21, 1.86	26.63	1.30, 1.99	28.36
13	1.82	43.59	1.60	45.50
14	------	51.51	------	51.03
15	1.11, 1.53	32.30	1.11, 1.53	32.30
16	1.42, 1.84	26.03	0.99, 1.82	26.46
17	2.08	46.22	2.20	45.63
18	1.00	15.92	1.00	15.92
19	0.89	17.10	0.89	17.10
20	------	79.40	------	81.98
21	------	180.68	------	179.28
22	1.90, 2.51	41.24	1.82, 2.21	39.05
23	5.20	75.35	5.36	76.38
24	5.31	124.91	5.21	123.67
25	------	140.36	------	141.30
26	1.78	25.72	1.80	25.72
27	1.76	18.11	1.78	18.26
28	1.01	28.52	1.01	28.52
29	0.85	16.95	0.85	16.95
30	0.92	16.65	0.94	16.43
	Ara		*Ara*	
1'	4.49	104.93	4.49	104.93
2'	3.87	75.06	3.87	75.06
3'	3.81	81.50	3.81	81.50
4'	3.95	68.62	3.95	68.62
5'	3.49, 3.87	64.52	3.49, 3.87	64.52
	Rha		*Rha*	
1''	5.19	101.85	5.19	101.85
2''	3.91	71.99	3.91	71.99
3''	3.70	71.99	3.70	71.99
4''	3.39	73.60	3.39	73.60
5''	3.86	70.09	3.86	70.09
6''	1.21	17.97	1.21	17.97
	Xyl		*Xyl*	
1'''	4.46	104.78	4.46	104.78
2'''	3.31	74.48	3.31	74.48
3'''	3.34	77.41	3.34	77.41
4'''	3.51	70.82	3.51	70.82
5'''	3.24, 3.89	66.72	3.24, 3.89	66.72

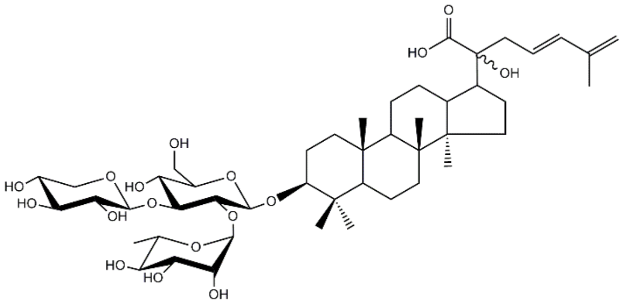

S18a. Structure of compound 29.

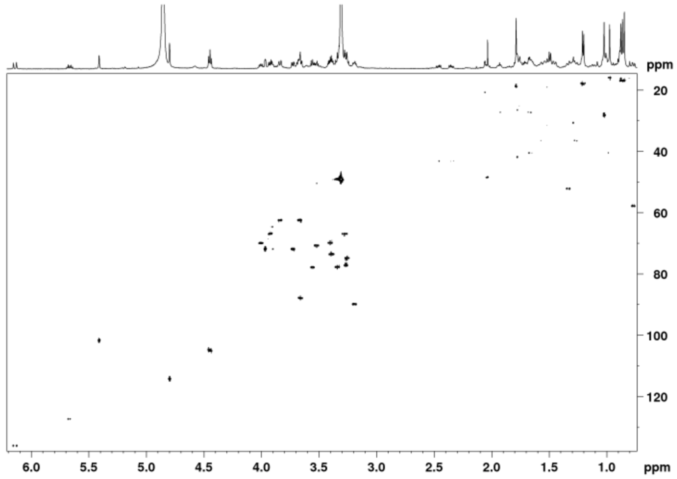

S18b. HSQC of compound 29.

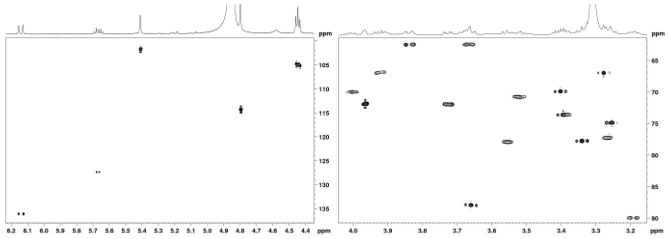

S18c. Selected region of HSQC showing anomeric and ringproton of the glycoside moiety of compound 29.

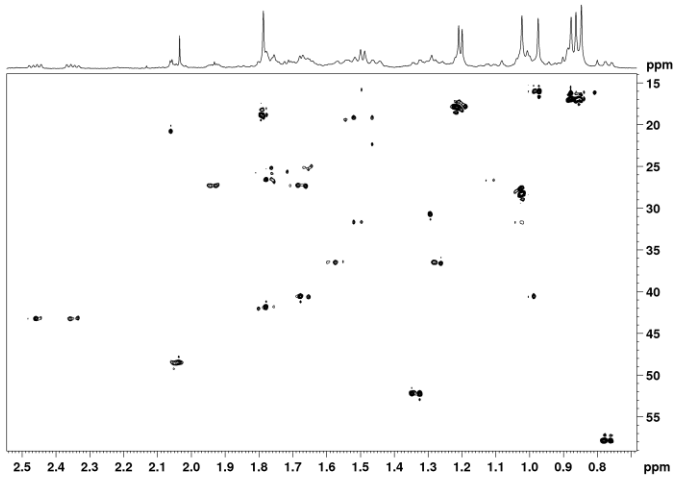

S18d. Selected region of HSQC showing the aglycone moiety of compound 29.

S18e. Chemical shifts of compound 29.

Pos.	29	
	δH	δC
1	0.99, 1.67	40.52
2	1.68, 1.93	27.26
3	3.19	90.00
4	------	40.36
5	0.77	57.87
6	1.46, 1.53	19.14
7	1.27, 1.57	36.49
8	------	41.70
9	1.33	52.22
10	------	38.03
11	1.21, 1.46	22.37
12	1.66, 1.76	25.13
13	1.78	41.84
14	------	51.33
15	1.03, 1.50	31.72
16	1.12, 1.77	26.60
17	2.04	48.41
18	0.97	15.92
19	0.86	16.93
20	------	80.03
21	------	181.21
22	2.34, 2.45	43.19
23	5.67	127.34
24	6.14	136.12
25	------	143.87
26	4.80, 4.80	114.31
27	1.79	18.84
28	1.02	28.30
29	0.85	16.94
30	0.88	16.79
	Glc	
1'	4.44	105.23
2'	3.55	77.94
3'	3.66	87.95
4'	3.40	69.94
5'	3.26	77.26
6'	3.67, 3.84	62.48
	Rha	
1''	5.41	101.71
2''	3.97	71.84
3''	3.73	71.99
4''	3.40	73.60
5''	4.01	69.41
6''	1.21	17.82
	Xyl	
1'''	4.45	104.79
2'''	3.26	74.92
3'''	3.34	77.70
4'''	3.52	70.82
5'''	3.28, 3.92	66.92

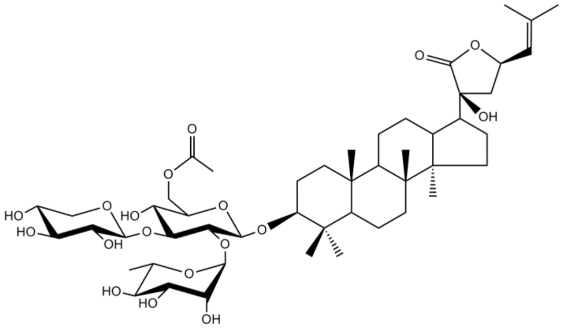

S19a. Structure of compound 31.

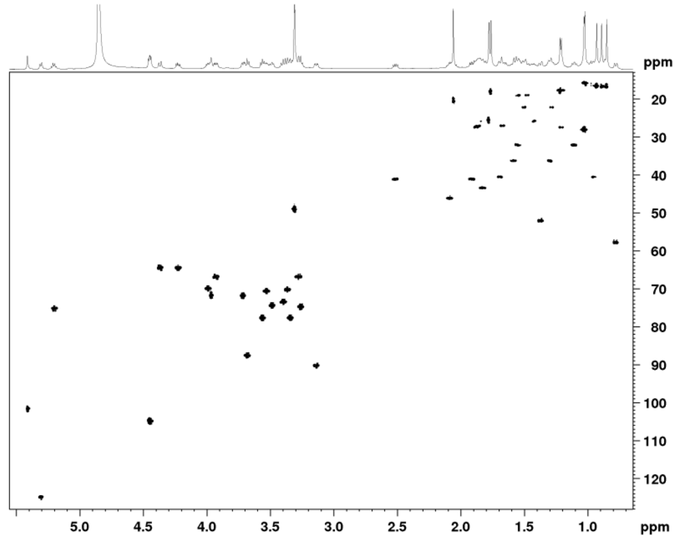

S19b. HSQC of compound 31.

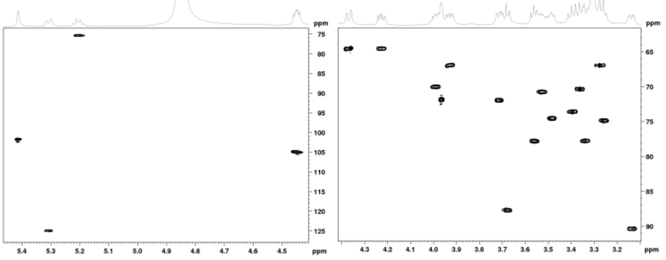

S19c. Selected region of HSQC showing anomeric and ringproton of the glycoside moiety of compound 31.

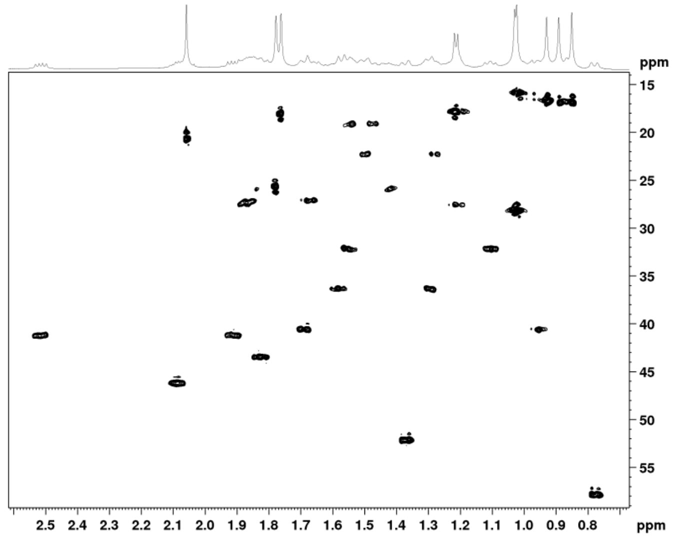

S19d. Selected region of HSQC showing the aglycone moiety of compound 31.

S19e. Chemical shifts of compound 31.

Pos.	31	
	δH	δC
1	0.96, 1.69	40.66
2	1.67, 1.86	27.18
3	3.14	90.29
4	------	40.38
5	0.78	57.93
6	1.47, 1.54	19.14
7	1.29, 1.58	36.41
8	------	41.78
9	1.37	52.23
10	------	37.79
11	1.28, 1.50	22.38
12	1.21, 1.89	27.63
13	1.82	43.59
14	------	51.52
15	1.10, 1.56	32.17
16	1.42, 1.84	26.01
17	2.08	46.22
18	1.02	15.91
19	0.89	16.94
20	------	79.60
21	------	180.53
22	1.92, 2.51	41.24
23	5.20	75.21
24	5.31	124.92
25	------	140.34
26	1.78	25.72
27	1.76	18.11
28	1.03	28.21
29	0.85	16.94
30	0.93	16.80
	Glc	
1'	4.44	104.93
2'	3.56	77.84
3'	3.68	87.66
4'	3.37	70.37
5'	3.49	74.48
6'	4.23, 4.37	64.52
-O**C**OCH3	------	172.48
-OCO***CH***3	2.06	20.74
	Rha	
1''	5.42	101.71
2''	3.97	71.84
3''	3.72	71.99
4''	3.40	73.60
5''	3.99	70.02
6''	1.22	17.82
	Xyl	
1'''	4.45	104.86
2'''	3.26	74.92
3'''	3.34	77.70
4'''	3.53	70.74
5'''	3.28, 3.93	66.87

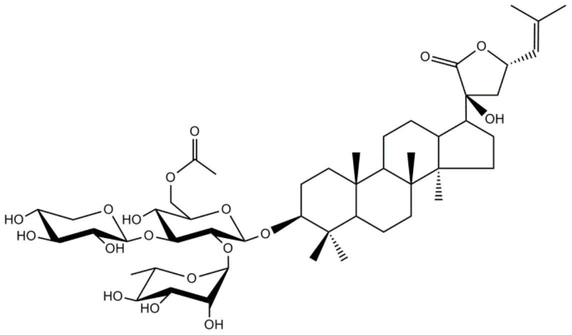

20a. Structure of compound 32.

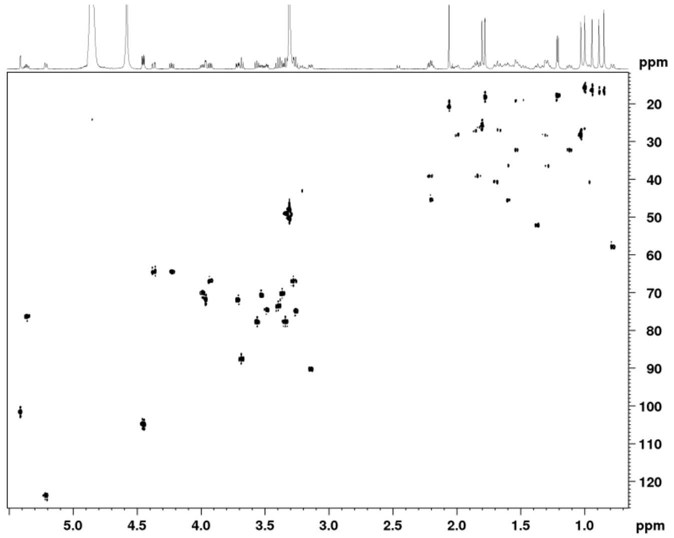

S20b. HSQC of compound 32.

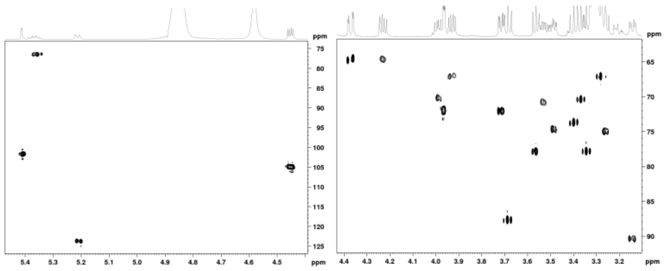

S20c. Selected region of HSQC showing anomeric and ringproton of the glycoside moiety of compound 32.

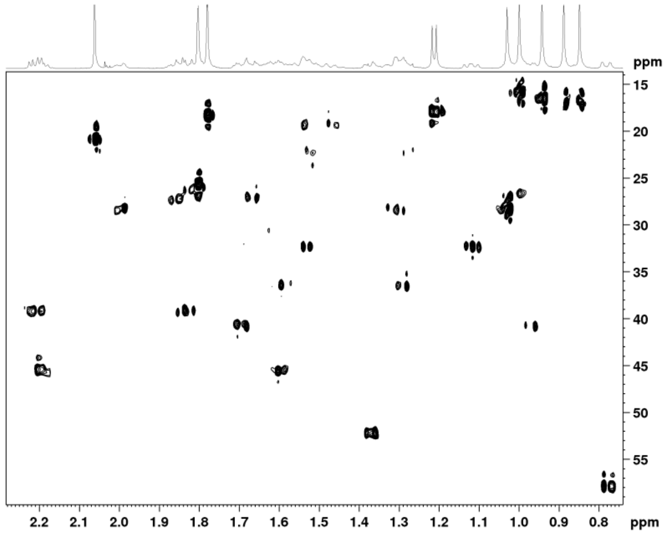

S20d. Selected region of HSQC showing the aglycone moiety of compound 32.

S20e. Chemical shifts of compound 32.

Pos.	32	
	δH	δC
1	0.96, 1.68	40.75
2	1.67, 1.86	27.28
3	3.15	90.25
4	------	40.07
5	0.78	57.90
6	1.48, 1.54	19.24
7	1.29, 1.60	36.40
8	------	41.40
9	1.38	52.13
10	------	37.55
11	1.28, 1.53	22.39
12	1.31, 2.00	28.42
13	1.61	45.48
14	------	50.79
15	1.11, 1.53	32.36
16	1.00, 1.83	26.34
17	2.20	45.66
18	0.99	15.82
19	0.89	16.99
20	------	81.50
21	------	179.17
22	1.84, 2.21	39.18
23	5.36	76.44
24	5.21	123.76
25	------	140.98
26	1.80	25.62
27	1.78	18.30
28	1.03	28.30
29	0.85	17.14
30	0.94	16.53
	Glc	
1'	4.44	104.94
2'	3.56	77.84
3'	3.69	87.80
4'	3.37	70.48
5'	3.49	74.68
6'	4.22, 4.37	64.54
-O***C***OCH3	------	172.24
-OCO***CH***3	2.06	20.79
	Rha	
1''	5.42	101.81
2''	3.96	71.89
3''	3.72	72.05
4''	3.40	73.64
5''	3.99	70.09
6''	1.21	17.86
	Xyl	
1'''	4.46	104.77
2'''	3.26	74.87
3'''	3.34	77.83
4'''	3.53	70.85
5'''	3.28, 3.93	67.00

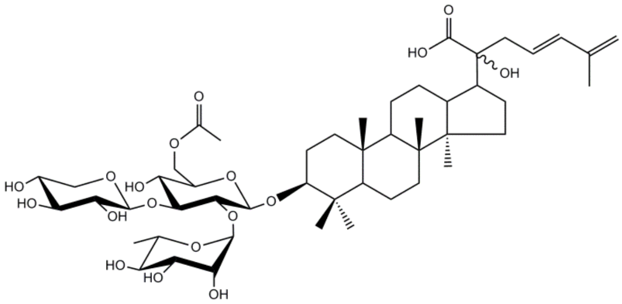

S21a. Structure of compound 34.

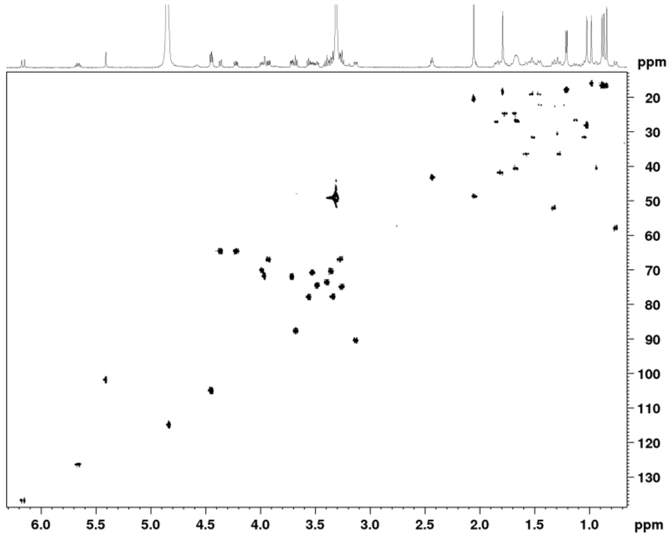

S21b. HSQC of compound 34.

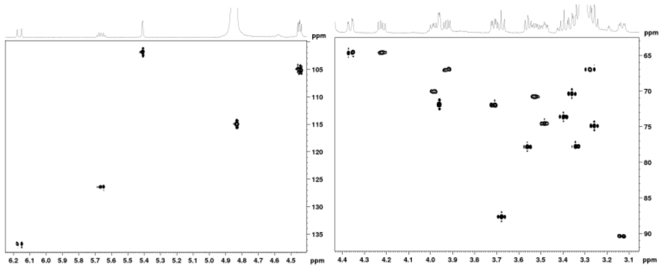

S21c. Selected region of HSQC showing anomeric and ringproton of the glycoside moiety of compound 34.

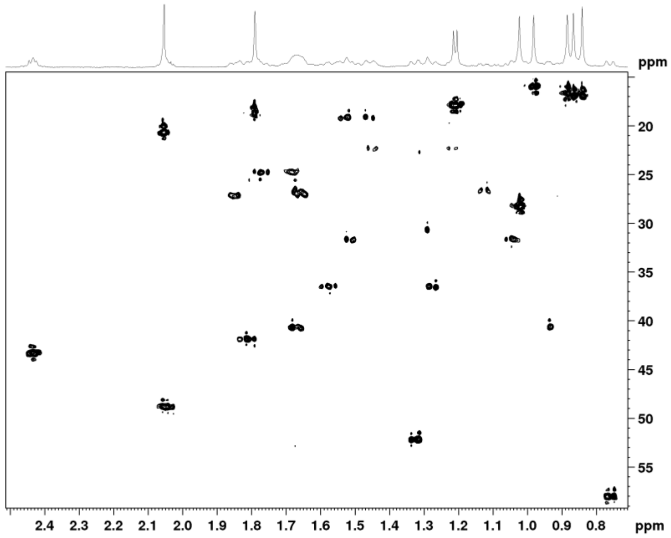

S21d. Selected region of HSQC showing the aglycone moiety of compound 34.

S21e. Chemical shifts of compound 34.

Pos.	34	
	δH	δC
1	0.94, 1.68	40.66
2	1.66, 1.85	27.27
3	3.13	90.44
4	------	40.22
5	0.76	58.01
6	1.46, 1.53	19.22
7	1.28, 1.58	36.49
8	------	41.73
9	1.33	52.16
10	------	37.79
11	1.22, 1.45	22.36
12	1.68, 1.78	24.77
13	1.82	41.90
14	------	51.32
15	1.06, 1.52	31.66
16	1.13, 1.66	26.61
17	2.05	48.78
18	0.98	15.98
19	0.87	17.01
20	------	79.61
21	------	180.11
22	2.43, 2.43	43.37
23	5.66	126.38
24	6.17	136.79
25	------	143.52
26	4.83, 4.84	114.97
27	1.79	18.77
28	1.03	28.43
29	0.85	17.01
30	0.89	16.72
	Glc	
1'	4.44	105.01
2'	3.56	77.92
3'	3.68	87.73
4'	3.36	70.45
5'	3.48	74.56
6'	4.23, 4.37	64.60
-O**C**OCH3	------	172.33
-OCO***CH***3	2.05	20.76
	Rha	
1''	5.41	101.79
2''	3.96	70.91
3''	3.72	72.06
4''	3.40	73.68
5''	3.99	70.16
6''	1.21	17.97
	Xyl	
1'''	4.45	104.87
2'''	3.26	75.00
3'''	3.34	77.77
4'''	3.53	70.90
5'''	3.27, 3.93	67.01

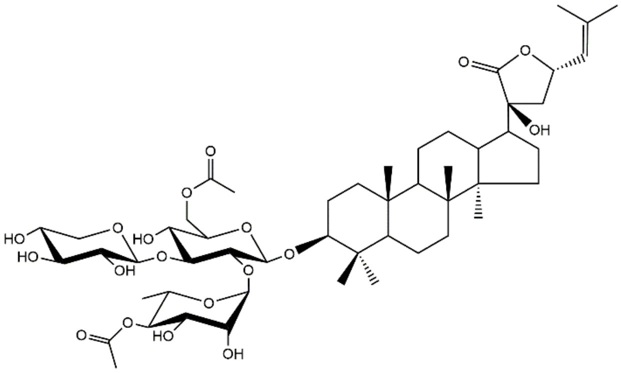

S22a. Structure of compound 39.

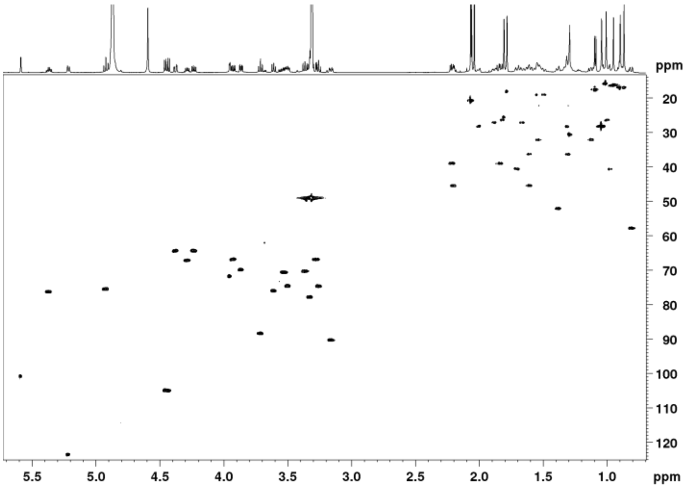

S22b. HSQC of compound 39.

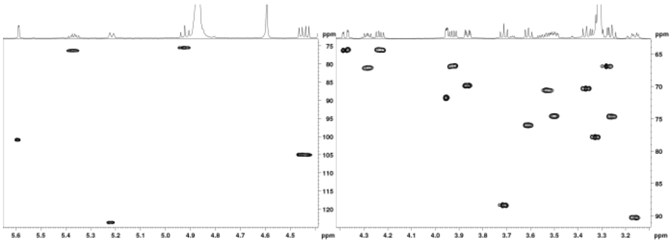

S22c. Selected region of HSQC showing anomeric and ringproton of the glycoside moiety of compound 39.

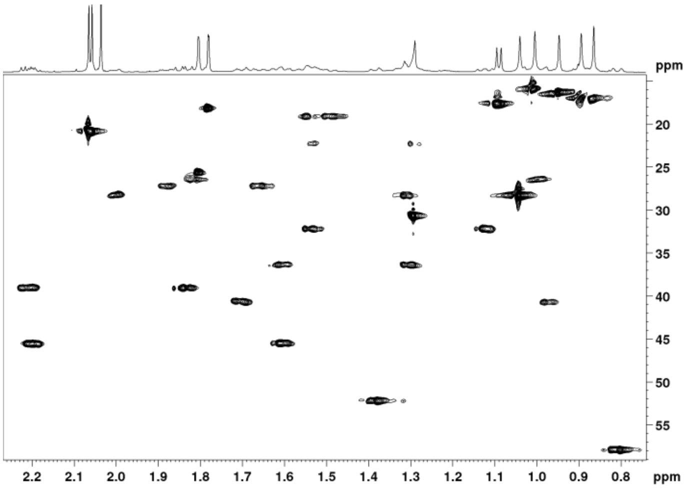

S22d. Selected region of HSQC showing the aglycone moiety of compound 39.

S22e. Chemical shifts of compound 39.

Pos.	39	
	δH	δC
1	0.97, 1.71	40.66
2	1.65, 1.87	27.19
3	3.16	90.43
4	------	40.24
5	0.81	57.93
6	1.50, 1.55	19.13
7	1.31, 1.61	36.41
8	------	41.63
9	1.39	52.22
10	------	38.12
11	1.30, 1.53	22.28
12	1.31, 2.00	28.36
13	1.61	45.49
14	------	51.03
15	1.13, 1.53	32.16
16	1.00, 1.82	26.46
17	2.20	45.57
18	1.00	15.92
19	0.89	17.02
20	------	81.74
21	------	179.27
22	1.84, 2.22	39.05
23	5.37	76.38
24	5.22	123.67
25	------	141.06
26	1.81	25.73
27	1.79	18.12
28	1.04	28.29
29	0.86	16.94
30	0.95	16.36
	Glc	
1'	4.44	105.06
2'	3.61	76.08
3'	3.71	88.53
4'	3.37	70.38
5'	3.50	74.69
6'	4.23, 4.38	64.44
-O**C**OCH3	------	172.48
-OCO***C**H*3	2.07	20.75
	Rha	
1''	5.59	100.98
2''	3.96	71.84
3''	3.87	69.94
4''	4.93	75.58
5''	4.29	67.23
6''	1.09	17.60
-O**C**OCH3	------	172.24
-OCO***C**H*3	2.07	20.75
	Xyl	
1'''	4.46	105.07
2'''	3.26	74.77
3'''	3.33	77.99
4'''	3.53	70.67
5'''	3.28, 3.93	67.00

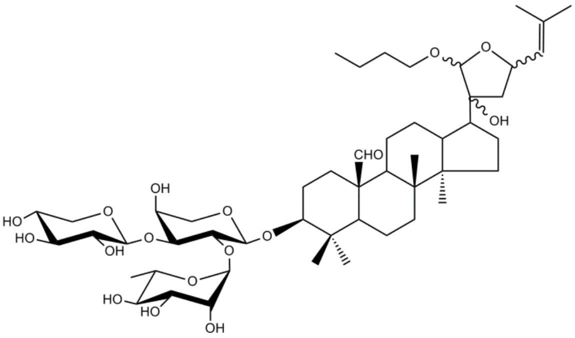

S23a. Structure of compound 41.

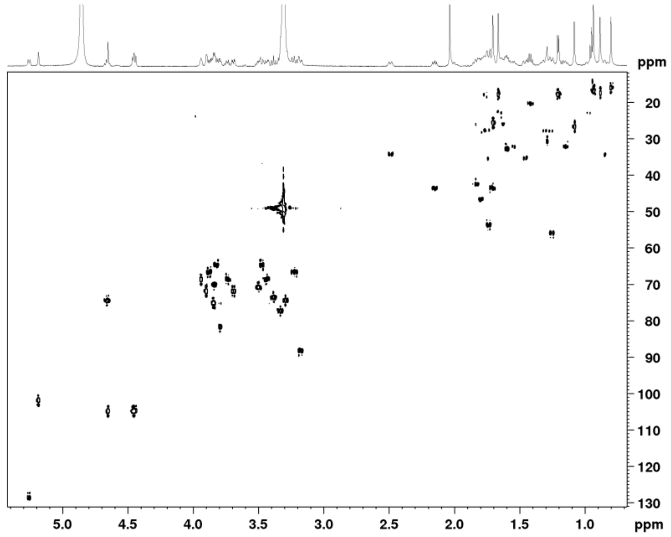

S23b. HSQC of compound 41.

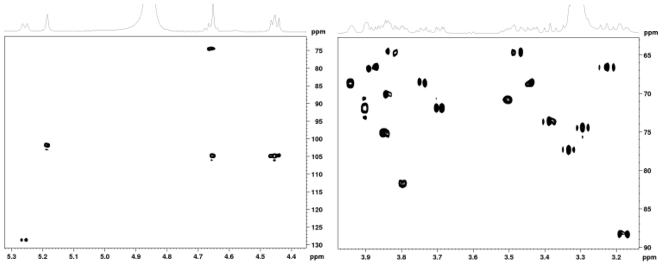

S23c. Selected region of HSQC showing anomeric and ringproton of the glycoside moiety of compound 41.

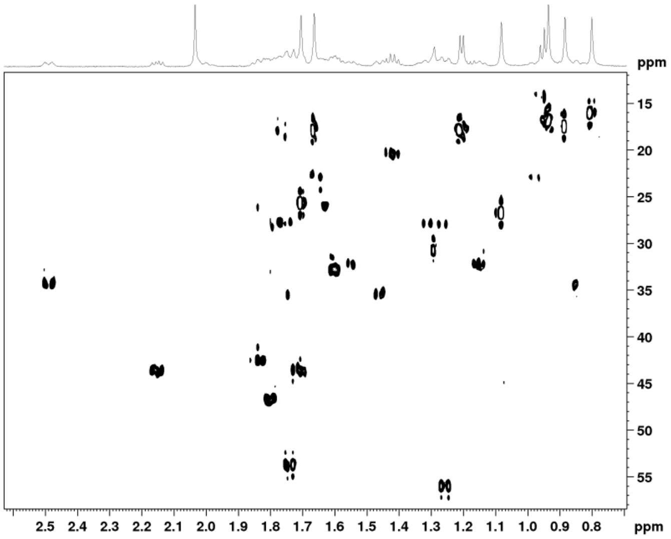

S23d. Selected region of HSQC showing the aglycone moiety of compound 41.

S23e. Chemical shifts of compound 41.

Pos.	41	
	δH	δC
1	0.85, 2.49	34.24
2	1.63, 1.84	26.04
3	3.18	88.25
4	------	40.78
5	1.27	55.88
6	1.78, 2.01	18.11
7	1.46, 1.75	35.42
8	------	41.64
9	1.74	53.70
10	------	53.85
11	0.98, 1.66	22.93
12	1.31, 1.75	27.76
13	1.84	42.43
14	------	51.30
15	1.15, 1.55	32.15
16	1.26, 1.78	27.94
17	1.80	46.67
18	0.89	17.46
19	10.18	207.86
20	------	83.27
21	4.65	104.80
22	1.71, 2.15	43.51
23	4.66	74.63
24	5.26	128.66
25	------	135.71
26	1.71	25.68
27	1.67	17.82
28	1.08	26.75
29	0.81	16.05
30	0.93	16.80
	Ara	
1'	4.46	104.92
2'	3.85	75.14
3'	3.80	81.81
4'	3.94	68.72
5'	3.47, 3.83	64.67
	Rha	
1''	5.19	101.86
2''	3.90	71.99
3''	3.69	71.85
4''	3.39	73.60
5''	3.84	70.09
6''	1.21	17.83
	Xyl	
1'''	4.44	104.65
2'''	3.29	74.48
3'''	3.33	77.33
4'''	3.50	70.81
5'''	3.22, 3.88	66.65
	n-butyl	
1'''	3.44, 3.74	68.60
2'''	1.60	32.75
3'''	1.43	20.30
4'''	0.95	14.22

S24. Purification of fraction by following isocratic elution, exception were made for fraction 20A and 20B.

Fraction	Isocratic method % ACN
6-7	30
8-16	35
17-18	40
19-24	45
25-29	50
30-34	55
35-39	60
41-42	70
